# Instruments for evaluating the quality of services in chronic diseases: scoping review*

**DOI:** 10.1590/1518-8345.7168.4293

**Published:** 2024-08-19

**Authors:** Gutembergue Santos de Sousa, Fabiane Verônica da Silva, Fabiana Gulin Longhi, Denise da Costa Boamorte Cortela, Pãmela Rodrigues De Souza Silva, Silvana Margarida Benevides Ferreira

**Affiliations:** 1 Universidade Federal do Mato Grosso, Cuiabá, MT, Brazil.; 2 Universidade de São Paulo, Biblioteca da Faculdade de Enfermagem, São Paulo, SP, Brazil.; 3 Universidade do Estado do Mato Grosso, Faculdade de Medicina, Cáceres, MT, Brazil.

**Keywords:** Validation Study, Evaluation of Research Programs, Surveys and Questionnaires, Chronic Disease, Health Services Research, Methods

## Abstract

**Objective:**

to map the scientific literature on the validity of instruments for evaluating the quality of services provided in primary health care for chronic diseases related to systemic arterial hypertension, diabetes *mellitus*, leprosy and tuberculosis.

**Method:**

scoping review, following the Joanna Briggs Institute method and described in accordance with the Preferred Reporting Items for Systematic Reviews and Meta-Analyses Extension for Scoping Reviews. 13 databases and gray literature were included. The selection of studies was carried out after removing duplicates and individual and paired evaluation. The data was extracted based on an elaborate script and presented in tables and charts.

**Results:**

the analysis of 28 selected studies showed that the majority were from Brazil, followed by China and Malaysia. Almost half of the validated instruments were generic, and the specific ones covered the evaluation of diabetes *mellitus* and leprosy. The types of validation carried out were content and construct.

**Conclusion:**

there is a need to construct specific instruments due to the scarcity of studies on the process of validating instruments for evaluating the quality of services provided by primary health care for chronic diseases.

## Highlights

**(1)** Validated instruments for evaluating services in chronic diseases.

**(2)** Possibility of carrying out new studies on adaptations of PCAT and PACIC.

**(3)** No evaluation was identified for minors under 18 years of age with chronic diseases.

**(4)** Use of a generic instrument to evaluate specific chronic diseases.

## Introduction

Chronic diseases are defined by the World Health Organization (WHO) as pathologies that develop slowly and persist for periods of more than six months, requiring more advanced therapy and care for a longer period of time, with these diseases being among the main causes of mortality and morbidity in the world^1^. 

The United Nations (UN) Sustainable Development Goals (SDGs) reinforce the need for greater attention to diseases such as diabetes, tuberculosis, arterial hypertension and leprosy, requiring greater efforts to prevent and treat these diseases, which are present in all regions of the world^2^.

In the Brazilian scenario, the Strategic Action Plan to Combat Chronic and Non-Communicable Diseases in Brazil 2021-2030 strengthens the guidelines for preventing these diseases and strengthening health promotion actions and reducing inequities and social inequalities, through the reorganization of health services, establishment of programs and projects, and investments in areas of knowledge that favor management research, innovation and the implementation of scientific evidence in services^3^.

Systemic arterial hypertension and diabetes *mellitus* are among the main chronic non-communicable diseases. The great impact of these diseases on the Brazilian population requires the adoption of effective measures and appropriate monitoring, in order to offer health care capable of acting adequately in interventions and that can result in a reduction in the incidence of these diseases, promoting activities related to prevention and timely pharmacological treatment^4^.

Leprosy and tuberculosis are chronic transmissible diseases present in Brazilian territory. These are infectious diseases that represent a major public health problem^5^. There is a regionalization factor related to these diseases, which presents different patterns of spatial distribution, linked to the increase in social inequalities and the existence of pockets of poverty. The behavior of these diseases can serve as an indicator of the development of a given region, reflecting the need to formulate public policies, basic sanitation, economic development and better access to health services^6^.

Therefore, leprosy and tuberculosis are priority diseases in the national scenario, with specific coordination that guides the entire prevention protocol, health promotion and care in Primary Health Care units, in addition to being diseases of interest for research throughout Brazil^5^.

Primary Health Care (PHC) is the priority and ordering service of the care network for the care of communicable and non-communicable chronic diseases. PHC, in Brazil, is based on the guiding principles of the *Sistema Único de Saúde* (SUS), and has the mission of offering actions and services within the concept of territoriality^7^. The quality of health and, mainly, of PHC services, is currently strengthening as it represents a preponderant political and social function, resulting in the process of transformation and appreciation of aspects and attributes capable of measuring the provision of services offered to the population^8^.

PHC, in this scenario, is characterized by a set of actions with the objective of developing comprehensive, quality care, which aims to increase people’s autonomy and their health situation individually and collectively, through inclusion in qualified health care services^9^.

In this aspect, the evaluation of health services is essential as a decision-making process based on scientific evidence capable of guiding and/or modifying the provision of services, ensuring an adequate response to the population’s health demands, which allows the reformulation of practices through managerial competence and the incorporation of information production to define new intervention strategies^10^.

From this perspective, the construction, adaptation and validation of service evaluation instruments is considered an essential management tool in health quality^11^. The validation of measurement instruments consists of creating forms and questionnaires that allow the measurement of what is proposed, as close as possible to reality, through psychometric properties and parameters that guarantee the validity and expanded use of these instruments^11^ ^-^ ^12^.

A preliminary search of PROSPERO (International Prospective Register of Systematic Reviews), MEDLINE (Medical Literature Analysis and Retrieval System Online), CDSR (Cochrane Database of Systematic Reviews) and JBI Evidence Synthesis (Joanna Briggs Institute) was conducted for verification, and no current or ongoing scoping review or systematic review was identified, requiring the need to conduct this study. 

Given the above, this study aimed to map the scientific literature on the validity of instruments for evaluating the quality of services provided in primary health care for chronic diseases related to systemic arterial hypertension, diabetes *mellitus*, leprosy and tuberculosis. 

This scoping review aims to corroborate, based on its findings, the knowledge available on the topic in question, so that more effective strategies can be developed to confront chronic diseases, based on the evaluation of the services provided in the primary health care, through instruments capable of guaranteeing the reliability and reproducibility of their use. 

## Method

### Type of study

This is a scoping review produced according to the criteria and recommendations established by the JBI^13^ and described in accordance with the Preferred Reporting Items for Systematic Reviews and Meta-Analyses extension for Scoping Reviews (PRISMA-ScR) checklist^14^. The protocol for this study is published in the Open Science Framework, at: https://osf.io/ynrht/. 

### Study scenario and information sources

The searches were carried out in the following databases: National Institutes of Health (PubMed), Cumulative Index to Nursing and Allied Health Literature (CINAHL), Cochrane Central, EMBASE, Scopus, Web of Science, Psychological Abstracts (PsyCINFO) and Leprosy Information Services (Infolep). The search strategy was adapted according to the protocols adopted by each database, using a combination of several descriptors. The search for gray literature was carried out in the Bank of Theses and Dissertations of the *Coordenação de Aperfeiçoamento de Pessoal de Nível Superior* (CAPES), DART - EBSCO Open Dissertation, Networked Digital Library of Theses and Dissertations (NDLTD), *Biblioteca Digital Brasileira de Teses e Dissertações* - (BDTD) - *Instituto Brasileiro de Informação em Ciência e Tecnologia* (IBICT) and American Chemical Society (ACS) Guide to Scholarly Communication.

### Period

This study was carried out from June to October 2023, following six steps: eligibility criteria, information sources, literature search, selection of sources of evidence, data extraction and analysis and presentation of data.

### Population

The review consisted of 4,083 studies, identified in the following databases: 2511 in PubMed, 152 in CINAHL, 305 in EMBASE, 324 in Scopus, 108 in *Web of Science*, 21 in PsycINFO, 37 in Infolep, 23 in Cochrane Library, 391 in *Catálogo de Teses e Dissertações CAPES*, 25 in DART - EBSCO Open Dissertation, 21 in Networked Digital Library of Theses and Dissertations (NDLTD), 153 in BDTD - *Biblioteca Digital Brasileira de Teses e Dissertações* - IBICT - *Instituto Brasileiro de Informação em Ciência e Tecnologia* and 12 in ACS Guide to Scholarly Communication. 

### Selection criteria

The research question was developed based on the acronym PCC (Population, Concept and Context)^15^, with P: P: Studies on validated instruments to measure the quality of health services; C: Validity process; and C: Care for systemic arterial hypertension, diabetes *mellitus*, leprosy and tuberculosis in primary health care. 

Experimental and quasi-experimental studies, methodological studies, analytical and descriptive observational studies, qualitative approaches, systematic reviews and meta-analyses, book chapters, conference abstracts, theses, dissertations and other sources of gray literature pertinent to the topic, such as journals and websites specialized in the health area, with no language restrictions and no time frame, were included. Studies that evaluated self-care and studies that were not available in full were excluded. Based on the acronym PCC, the research question was formulated: What evidence is available in the literature on instruments for evaluating the quality of services provided in primary health care for chronic diseases? 

Research, in this scenario, into leprosy, tuberculosis, arterial hypertension and diabetes *mellitus* is justified because these diseases are of interest to Brazilian health policies, having their own specific actions and guidelines for all aspects of prevention and health promotion, in health care networks, from the Brazilian *Ministério da Saúde* (Ministry of Health).

### Study variables

A specific instrument was developed to collect data from articles in the best possible way. This instrument was composed of the following variables: type of journal, country in which the study was carried out, chronic disease assessed by the instrument, population studied, type of validity adopted, type of scale, content validity technique, construct validity technique, calculation of internal consistency and calculation of reliability and identification of the main findings in the set of evidence.

### Data collection

The search strategy followed three steps^16^. Firstly, a preliminary search was carried out in the PubMed databases and in the Cumulative Index to Nursing and Allied Health Literature (CINAHL), with the aim of identifying the terms contained in titles, abstracts and descriptors to assemble the search strategy. In step two, a second search was carried out using the terms found in the first step added to the identified descriptors. In the third step, the researchers searched the reference lists for studies not retrieved in the first two steps. 

A combination of the following descriptors was used: Pessoal de Saúde / Health Personnel / Personal de Salud OR Gestor de Saúde / Health Manager / Gestor de Salud OR Pacientes / Patients / Pacientes AND Estudo de Validação / Validation Study / Estudio de Validación AND Qualidade da Assistência à Saúde / Quality of Health Care.

The complete search strategy is described in Figure 1, updated on 08/22/2023.

**Figure 1:** Search strategy according to databases. Cuiabá, MT, Brazil, 2023

Data base	Search strategy
PubMed	((“Health Personnel”[Mesh] OR “Health Personnel”) OR (“Health Manager”) OR (“Patients”[Mesh] OR Patients OR Patient)) AND ((“Validation Study” OR “Validation Studies” OR “Validation Tool” OR “Validation Tools” OR “Validation Instruments” OR “Validation Instrument”) AND ((“Health Evaluation”) OR (“Quality of Health Care”[Mesh] OR “Quality of Health Care”))) AND (“Primary Health Care”[Mesh] OR “Primary Health Care” OR “Primary Healthcare” OR “Primary Care”)
CINAHL	(((MH “Health Personnel”) OR “Health Personnel”) OR ((MH “Patients”) OR (Patient OR Patients))) AND ((MH “Validation Studies”) OR (“Validation Study” OR “Validation Studies” OR “Validation Tool” OR “Validation Tools” OR “Validation Instruments” OR “Validation Instrument”)) AND ((“Health Evaluation”) OR ((MH “Quality of Health Care”) OR “Quality of Health Care”)) AND ((MH “Primary Health Care”) OR “Primary Health Care”)
EMBASE	(‘health care personnel’/exp OR ‘health care personnel’ OR ‘patient’/exp OR patient) AND (‘validation study’/exp OR ‘validation study’) AND (‘health care quality’/exp OR ‘health care quality’ OR ‘health evaluation’) AND (‘primary health care’/exp OR ‘primary health care’)
Scopus	TITLE-ABS-KEY ((“Health Personnel” OR “Health Manager” OR Patient) AND (“Validation Study” OR “Validation Studies” OR “Validation Tool” OR “Validation Tools” OR “Validation Instruments” OR “Validation Instrument”) AND (Evaluation OR “Quality of Health”) AND (“Primary Health Care” OR “Primary Healthcare” OR “Primary Care”))
Web of Science	(“Health Personnel” OR “Health Manager” OR Patient) AND (“Validation Study” OR “Validation Studies” OR “Validation Tool” OR “Validation Tools” OR “Validation Instruments” OR “Validation Instrument”) AND (Evaluation OR “Quality of Health”) AND (“Primary Health Care” OR “Primary Healthcare” OR “Primary Care”)
PsycINFO	(“Health Personnel” OR “Health Manager” OR Patient) AND (“Validation Study” OR “Validation Studies” OR “Validation Tool” OR “Validation Tools” OR “Validation Instruments” OR “Validation Instrument”) AND (Evaluation OR “Quality of Health”) AND (“Primary Health Care” OR “Primary Healthcare” OR “Primary Care”)
Infolep	(“Validation Study” OR “Validation Studies” OR “Validation Tool” OR “Validation Tools” OR “Validation Instruments” OR “Validation Instrument”)
Cochrane Library	(“Patients” OR “Health Personnel” OR “Health Manager”) AND (“Validation Study” OR “Validation Studies” OR “Validation Tool” OR “Validation Tools” OR “Validation Instruments” OR “Validation Instrument”) AND (“Quality of Health Care” OR Evaluation) AND (“Primary Health Care”) = in Title Abstract Keyword
CAPES *Catálogo de Tteses e Dissertações*	Validação AND Avaliação AND “Atenção Primária à Saúde”
DART - EBSCO Open Dissertation	(“Health Personnel” OR “Health Manager” OR Patient) AND (“Validation Study” OR “Validation Studies” OR “Validation Tool” OR “Validation Tools” OR “Validation Instruments” OR “Validation Instrument”) AND (“Primary Health Care” OR “Primary Healthcare” OR “Primary Care”)
Networked Digital Library of Theses and Dissertations (NDLTD)	(“Health Personnel” OR “Health Manager” OR Patient) AND (“Validation Study” OR “Validation Studies” OR “Validation Tool” OR “Validation Tools” OR “Validation Instruments” OR “Validation Instrument”) AND (Evaluation OR “Quality of Health”) AND (“Primary Health Care” OR “Primary Healthcare” OR “Primary Care”)
BDTD - *Biblioteca Digital Brasileira de Teses e Dissertações* - IBICT - *Instituto Brasileiro de Informação em Ciência e Tecnologia*	Validação AND Avaliação AND “Atenção Primária à Saúde”
*ACS* Guide to Scholarly Communication	(“Health Personnel” OR “Health Manager” OR Patient) AND (“Validation Study” OR “Validation Studies” OR “Validation Tool” OR “Validation Tools” OR “Validation Instruments” OR “Validation Instrument”) AND (“Primary Health Care” OR “Primary Healthcare” OR “Primary Care”)

### Data extraction

The studies were selected by two independent reviewers, with experience in the topic and in carrying out scoping reviews. A third reviewer was used to resolve disagreements regarding study selection, opting for their inclusion or exclusion.

The first selection occurred by reading the title and summary, taking into account the presence of elements that indicated or not that it was an evaluative study, according to the diseases listed in the criteria. Then, the texts were read in full and evaluated according to the inclusion/exclusion criteria. Texts from gray literature had the same evaluation process. 

Data extraction occurred in accordance with the guidelines established in the JBI manual^13^. JBI is an institution that establishes standards for carrying out systematic reviews, updating data and making recommendations necessary for the quality of these products^12^.

### Data processing

The results obtained were imported into the EndNote Web program, where the investigation of the duplicity of bibliographic references was carried out^17^. For analysis, selection and exclusion of studies, the Rayyan (Qatar Computing Research Institute, Doha, Qatar)^18^ software was used, with the same selection and exclusion criteria listed previously, since the platform allowed migrating the file of databases, making data processing easier and more agile through this tool.

Data processing occurred in accordance with the guidelines established in the JBI manual^13^. After selecting the final sample, the results were organized through charts, figures and descriptive tables.

### Ethical aspects

As this is a study with data in the public domain and available in the literature, there was no need for consideration by a Research Ethics Committee. However, it should be noted that copyright was respected with due citations to each author and periodical. 

## Results

**Figure 2:**
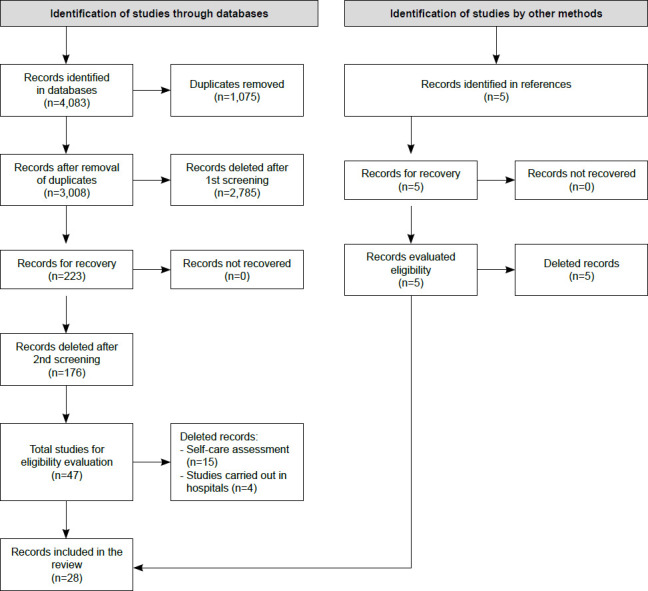
Summary of the steps of the systematic scoping review according to the adaptation of the Preferred Reporting Items for Systematic Reviews and Meta-Analyses. Cuiabá, MT, Brazil, 2023

In the 13 databases, 4,083 studies were identified, 2511 of which were in MEDLINE/PubMed, 152 in CINAHL, 305 in EMBASE, 324 in Scopus, 108 in Web of Science, 21 in PsycINFO, 37 in Infolep, 23 in Cochrane Library, 391 in *Catálogo de Teses e Dissertações* CAPES, 25 in DART - EBSCO Open Dissertation, 21 in Networked Digital Library of Theses and Dissertations (NDLTD), 153 in BDTD - *Biblioteca Digital Brasileira de Teses e Dissertações* - IBICT - *Instituto Brasileiro de Informação em Ciência e Tecnologia* and 12 in ACS Guide to Scholarly Communication. After selection by title, abstract and application of eligibility criteria, 28 studies^19^ ^)-(^ ^46^ comprised the final sample, according to the flowchart (Figure 2).

There was a higher percentage of studies published without data registration regarding the data collection period (n=15; 53.6%). The studies recording the data collection period were carried out in 2003 and 2016 (n=13; 46.4%). As for the year of publication, 6 (21.5%) studies were published between 2004 and 2010, 19 between 2011 and 2020 (67.8%) and 3 (10.7) were published after 2020.

Regarding the data collection period, it was observed that a large part of the articles did not contain this information, a fact that could harm the assessment of their methodological quality. Most of the articles were published before 2020, which indicates that their production goes back to this date, due to the period of evaluation of the articles by the scientific journal. Both facts deserve to be highlighted, as they can be considered a limitation when evaluating the quality of this product and the manuscripts used.

Concerning the type of periodical, most studies were published in international journals (n=22; 78.6%). Among the countries with the highest publications are Brazil (n=7; 25.0%), followed by China (n=3; 10.7%) and Malaysia (n=3; 10.7%). The studies presented at least two or three different types of validation instrument, most of which were generic questionnaires (n=13, 46.4%). The questionnaires were for assessments of chronic diseases in a general context, followed by instruments for evaluating diabetes *mellitus* and leprosy (n=5; 17.9%). In this scoping review, no instruments were identified for evaluating the quality of services provided in chronic diseases for the population of children and adolescents (< 18 years old). The majority of studies (n=19; 68%) were carried out in adult and elderly populations (> 18 years old).

Regarding the type of validation studies identified, cross-cultural adaptation was found in 50% (n=14) of the studies; 19 (67.9%) carried out content validity; 18 (64.3%), semantic validity; and 23 (82.1%), construct validity. Internal consistency was calculated in 92.8% (n=26) of the study sample, and reliability was calculated through test/retest in only 13 (46.4%) studies. Regarding the type of scale adopted, most studies opted for the 5-point Likert scale (n=16; 57.1%). The above data are described in Table 1.

**Table 1:** Characterization of scientific production on instruments for evaluating health services for chronic diseases in Primary Health Care (n = 28). Cuiabá, MT, Brazil, 2023

Variables	n*	%^†^
**Type of publication journal**		
National	06	21.4
International	22	78.6
**Country where the study was carried out**		
Germany	02	07.1
Brazil	07	25.0
China	03	10.7
France	02	07.1
Netherlands	02	07.1
Malaysia	03	10.7
Others^‡^	09	32.3
**Chronic disease evaluated by the survey instrument**		
Generic instrument^§^	13	46.4
Hypertension	03	10.7
Diabetes	05	17.9
Leprosy	05	17.9
Tuberculosis	02	07.1
**Population studied**		
< de 18 years old	00	00.0
> de 18 years old	19	68.0
Health professionals	07	25.0
Managers	02	07.0
**Type of validity adopted** ^||^		
Cross-cultural adaptation	14	50.0
Content validity	19	67.9
Face validity	06	21.4
Semantic validity	18	64.3
Construct validity	23	82.1
**Calculation of the internal consistency of the instrument**		
Yes	26	92.9
No	02	07.1
**Calculation of instrument reliability**		
Yes	13	46.4
No	15	53.6
**Type of scale adopted**		
Dichotomous	01	3.6
3-point Likert	01	3.6
4-point Likert	04	14.3
5-point Likert	16	57.1
Others^¶^	06	21.4
**Identified instruments**		
PACIC** adaptations	08	28.5
PCAT^††^ adaptations	08	28.5
Other miscellaneous instruments	12	43.0

*N = Absolute number; ^†^% = Percentage; ^‡^Countries with only one published study: Canada, Denmark, Spain, Ethiopia, Finland, Netherlands, Turkey, Thailand and one not described; ^§^Used to evaluate chronic diseases in general, without specification; ^||^Most studies adopt more than one type of validity; ^¶^Did not mention the type of scale/11-point Likert; **PACIC = Patient Assessment of Chronic Illness Care (PACIC); ^††^PCAT = Primary Care Assessment Tool

Regarding the use of instruments, similar percentages of adaptations were observed for both the Patient Assessment of Chronic Illness Care (PACIC) (n=8; 28.5%) and the Primary Care Assessment Tool (PCAT); 43% (n=12) dealt with instruments other than those highlighted as having a large percentage. With regard to content validity, there was a higher percentage of use of the focus group technique to analyze the instrument questions (n=11; 57.9%). In construct validity studies, 20 of them performed factor analysis (n=23;82.1%), followed by the convergent construct technique (n=7;30.4%).

As for diseases, higher percentages were observed for the instruments for diabetes *mellitus* (n=5;17.9%) and leprosy (n=5;17.9%). The main findings for the instrument on diabetes *mellitus* were: originality and transcultural adaptation; continuity of care, prevention and health promotion as phenomena of interest; target population adults/elderly and healthcare professionals; divergent and convergent construct validity technique; satisfactory calculation of Cronbach’s alpha as a measure of internal consistency and psychometric properties considered valid, being able to be used in scientific studies.

The main findings for the leprosy instrument were: adaptation of the PCAT; target population adults, community health agents, health professionals and managers; semantic validity and construct validity mirroring another instrument of its kind; calculation of reliability through test/retest and psychometric properties considered valid and suitable for use in scientific studies. These findings are available in Figure 3.

**Figure 3:** Synthesis of the main evidence found in validation studies of instruments for evaluating diabetes and leprosy services (n = 10). Cuiabá, MT, Brazil, 2023

Chronic disease	Main findings
Diabetes	Instruments originally developed^20^ ^),(^ ^25^ and cross-culturally adapted^19^ ^),(^ ^30^ ^),(^ ^37^.
	Presented continuity of care^19^ ^),(^ ^25^, prevention and health promotion^20^ ^),(^ ^30^ and care^37^ as phenomena of interest.
	Target audience and study participants were adult population^25^, adult and elderly population^19^ ^,^ ^37^ and health professionals^20^ ^),(^ ^30^.
	Used the convergent/divergent construct validity technique^20^ ^),(^ ^25^ ^),(^ ^37^.
	Presented Cronbach’s alpha greater than 0.8^19^ ^),(^ ^25^ ^),(^ ^30^ ^),(^ ^37^.
	The instruments presented psychometric properties considered valid, being suitable for use in scientific studies^19^ ^)-(^ ^20^ ^),(^ ^25^ ^),(^ ^30^ ^),(^ ^37^.
Leprosy	The instruments were adapted from the original instrument called *Primary Care Assessment Tool* ^42^ ^)-(^ ^45^.
	The studies were carried out in the adult population^40^ ^),(^ ^44^, with Community Health Agents^43^, with health professionals^42^ and with managers^45^.
	Semantic validity and evaluation of questions regarding clarity, understanding and adequacy of items were carried out^40^ ^),(^ ^42^ ^)-(^ ^45^.
	The instruments had their construct evaluated through factor analysis^43^ or the mirror validity technique with another instrument^42^ ^),(^ ^44^ ^)-(^ ^45^.
	The reliability of the instrument was calculated through test/retest^40^ ^),(^ ^42^ ^)-(^ ^45^.
	The instruments presented psychometric properties considered valid, being suitable for use in scientific studies^40^ ^),(^ ^42^ ^)-(^ ^45^.

## Discussion

The present study identified a lack of studies that dealt with the process of validating instruments for evaluating the quality of services provided by PHC in chronic diseases in a general context, which includes evaluation instruments regarding problems such as arterial hypertension, diabetes, leprosy and tuberculosis, which was the scope of investigation of this review. 

Most studies were published in international journals; produced mainly in Brazil, China and Malaysia; the majority of validated research instruments were generic instruments, followed by specific instruments for diabetes and leprosy; the studies were carried out on a population over 18 years old, with content validity and construct validity as the main validation processes; there was an internal consistency calculation in most studies and reliability was not calculated in any of the studies; most instruments had a 5-point Likert scale, where content validity occurred mainly through the focus group technique and construct validity through factor analysis; the main instruments found were adaptations of the PACIC and PCAT.

Regarding the publication of studies in international periodicals (publications carried out in journals in other countries outside Brazil), there are classification criteria to define international circulation. Therefore, the process of internationalization of scientific production cannot be characterized based solely on the country. This discussion also involves the indexing of the journal in international databases and the quality of evidence in the scientific world^47^.

The incessant search for product quality and the use of the appropriate method in scientific studies is increasingly escalating. Thus, as internationalization spreads and begins to be seen as a goal and objective to be achieved by authors and institutions, journals indexed in recognized international databases and with a high impact factor have more visibility and prestige to attract and select good studies^48^.

When one observes that most of the studies found were produced in Brazil, it is necessary to reflect on the significant increase in vacancies and master’s and doctorate programs in the country, generating researches that need to use reliable questionnaires, and which generally use validated instruments or carrying out validation studies^49^. Health research in Brazil has been building spaces aimed at the search for quality and improvement of techniques and enhancements in scientific studies, focusing mainly on the use of technologies and evaluations of services from different perspectives^50^.

Caring for patients with chronic diseases is one of the main challenges in today’s world. It is notable that a patient with a chronic condition accesses health services more than patients who do not have chronic conditions. Given these facts, the importance of validating generic instruments for evaluating the quality of care provided to patients with chronic diseases in PHC is highlighted.

It is also worth highlighting the important central role of PHC in the SUS, as it is a strong and consolidated care model compared to models from other countries. Parallel to this, the increase in Primary Care and ESF coverage over recent years in Brazil may also be a contributing factor to the growing demand for healthcare based on scientific evidence and for the creation of care and management protocols within PHC, encouraging the production of technologies and knowledge in this area^7^.

Assessment in leprosy is extremely important to measure aspects that may impact the quality of life of patients and the quality of services provided, in addition to being a tool for monitoring and feedback on the functioning of the actions and perceptions of service users^51^. Therefore, leprosy remains one of the priority diseases in the country’s public health policies.

Diabetes is one of the main chronic diseases that affect the world population and is linked to several biological and behavioral factors. It is also one of the main causes of death from chronic diseases^4^. These causes strengthen the need to carry out scientific studies based on these problems and, with this, the use of validated data collection tools capable of actually measuring what is proposed.

Regarding the fact that most of the research instruments in the validation studies included target the adult and elderly population, it is known that age is an important risk factor for some existing chronic diseases^52^, which can justify the finding in question in this review. Brazil is an aging country with an imminent risk of older people who require greater health care and support from the social protection network^53^, highlighting the real need to carry out research that impacts the cycle of chronic diseases in these age groups.

It is noteworthy that no instruments were found to assess chronic diseases in the age group of children and adolescents in a generic way, and specifically for the diseases listed in this review, based on the eligibility criteria adopted. The presence of chronic diseases in these age groups can serve as an evaluation parameter for the quality and organization capacity of health networks within an integrated system^54^.

At this point, health programs aimed at children and adolescents in PHC have been showing growth and a search to bring this clientele closer to health services, requiring mechanisms that seek to evaluate the performance of health actions and services made available to this target audience. 

The instrument content validation process is essential in the process of construction and selection of the topics covered, seeking to make the content more reliable and improve the measuring instrument, so that it can truly represent the theory that supported the construction step and measure how much these items are capable of reproducing the proposed phenomenon^55^. Given this, the fact that most of the studies identified in this review have passed through the content validity step may suggest greater value to the instrument produced. It is also worth highlighting that studies that carried out cross-cultural adaptation do not mention the content validity step; however, the original instrument possibly went through this step.

It was observed that in the majority of studies that described having used content validity, this occurred through the focus group technique. This technique is similar to a group interview, where, based on the inclusion and exclusion criteria, experts (specialists) are selected and meet to evaluate the issues present in the measurement instrument and seek a group consensus^56^.

Most of the studies selected in this review underwent construct validity using the factor analysis technique. Construct validity is a property adopted in psychometric tests to assess whether the measuring instrument is truly capable of measuring what it is intended to measure, thus making it possible to determine characteristics capable of explaining the variances and the real meaning of the test^57^.

As for the construct, it is necessary to draw attention to the few constructs/domains related to the mental health of the target audience for service evaluation. The aspects inherent to mental health constitute a robust health policy for users as well as professionals and managers, with growth that deserves to be highlighted through its care and management protocols.

The use of factor analysis in construct validity provides evaluative support for possible correlations between variables and their relations with each other, defined through factors, where a smaller number of latent traits can explain a greater number of variables^57^ ^)-(^ ^58^. Therefore, studies that present the construct validity process, theoretically, have greater robustness of valid psychometric properties, better representing the phenomenon studied, and being able to translate the desired reality as closely as possible.

The convergent/divergent construct refers to a technique in which other instruments are used that measure the same theoretical construct and, thus, it is assessed whether both instruments will present inversely or directly proportional quantities, allowing validation based on theoretical concepts^59^. Mirror validation was used as a technique through which a set of instruments was adapted from the PCAT, and factor analysis was carried out on one of them, and on the others, since it was not possible to perform factor analysis due to the size of the sample, mirror validation was carried out, using as reference the instrument in which the factor analysis took place^43^ ^),(^ ^45^ ^)-(^ ^46^.

Regarding the calculation of internal consistency, only two studies did not mention carrying out such a test. Internal consistency indicates whether an instrument is homogeneous or not. It expresses whether the subparts of the instrument are capable of portraying the measurement of the same inherent characteristic. A high internal consistency may indicate that the items of an instrument measure the same theoretical construct^57^ ^)-(^ ^58^. Therefore, the calculation of internal consistency is essential in the process of validating measurement instruments.

In this scoping review, it was observed that the majority of validated instruments did not mention calculating reliability through test/retest. This calculation measures the temporal stability of the questionnaire and its ability to reproduce the same result over time and space^57^ ^)-(^ ^58^. In view of this, it is necessary and important to adopt this psychometric measure in test validation, contributing to a higher quality of the measuring instrument, as long as this instrument accepts this type of measure, since this psychometric measure may not be adequate, due to the characteristics of the instrument, the type of population involved or the effects of time.

In the studies listed, the PACIC was the most adapted instrument. This is an instrument that has been tested in several countries around the world, based on the Chronic Care Model (CCM), and supported by evidence from many studies^60^. The instrument can be used generically or for specific chronic diseases, showing satisfactory results in the evaluation of services, being considered easy to apply and low cost^60^.

The PCAT, in turn, was proposed and validated by a team led by Barbara Starfield, in the United States of America, as a psychometric scale to evaluate, through scores, all attributes of PHC^9^. From then on, several Brazilian authors and those from other countries began to use the PCAT as an instrument for assessing PHC, and to carry out cross-cultural adaptation processes of the generic instrument and specific adaptations for some chronic diseases in particular^61^.

Continuity of care reflects the concepts of the existence of longitudinality, ensuring care in a timeline, as many times as necessary, dimensioning links between prevention and health promotion actions, also reflecting issues relating to access to health services^62^. In chronic diseases, continuity of care, prevention and health promotion are extremely important as tools for caring for the user and for properly directing actions in PHC.

The limitations of the study refer to the lack of clarity in the description of some validation processes between the instruments in the selected studies, which may cause possible bias in the results presented. The majority of studies were published before 2020, which indicates that their production goes back to this date, due to the period of their evaluation by the scientific journal, which also leads to a limitation when evaluating the quality of this product and the manuscripts used.

## Conclusion

The main findings refer to the predominance of validation of generic and specific instruments for leprosy and diabetes, which constitute a priority in primary health care for chronic diseases, and a knowledge gap was highlighted for validated instruments for care in children and adolescents. Most of the validated instruments were adaptations of the PACIC and PCAT, demonstrating the importance of these instruments in the evaluation of chronic diseases in the adult and elderly population and based on the perception of health professionals and managers.

The evidence indicates a lack of construction of specific instruments, and an absence of studies in the child and adolescent population on the process of validating instruments for evaluating the quality of services provided by primary health care in chronic diseases listed in the review.

Therefore, it is recommended that new studies be carried out, and even the validation of specific instruments to evaluate the quality of services given the chronicity of these diseases in the population of children and adolescents.
