# Supplementary material for: Instruments for evaluating the quality of services in chronic diseases: scoping review
Source: Rev Lat Am Enfermagem. 2024 Aug 19;32:e4293. doi: 10.1590/1518-8345.7168.4293 (PMC11335071; doi:10.1590/1518-8345.7168.4293)
Supplement: Translation

# Translation: Instrumentos para avaliação da qualidade de serviços em doenças crônicas: revisão de escopo*

**DOI:** 10.1590/1518-8345.7168.4294

**Authors:** Gutembergue Santos de Sousa, Fabiane Verônica da Silva, Fabiana Gulin Longhi, Denise da Costa Boamorte Cortela, Pãmela Rodrigues De Souza Silva, Silvana Margarida Benevides Ferreira

**Affiliations:** 1 Universidade Federal do Mato Grosso, Cuiabá, MT, Brasil.; 2 Universidade de São Paulo, Biblioteca da Faculdade de Enfermagem, São Paulo, SP, Brasil.; 3 Universidade do Estado do Mato Grosso, Faculdade de Medicina, Cáceres, MT, Brasil.

**Keywords:** Estudo de Validação, Avaliação de Programas e Instrumento de Pesquisa, Inquéritos e Questionários, Doença Crônica, Pesquisa sobre Serviços de Saúde, Método

## Abstract

**Objetivo:**

mapear a literatura científica sobre a validação de instrumentos para a avaliação da qualidade dos serviços prestados na atenção primária à saúde em doenças crônicas relacionadas com hipertensão arterial sistêmica, diabetes *mellitus*, hanseníase e tuberculose.

**Método:**

revisão de escopo, seguindo o método do Instituto Joanna Briggs e descrito de acordo com o *Preferred Reporting Items for Systematic Reviews and Meta-Analyses Extension for Scoping Reviews*. Foram incluídas 13 bases de dados e literatura cinzenta. A seleção dos estudos foi realizada após a remoção de duplicatas e avaliação individual e em pares. Os dados foram extraídos com base em um roteiro elaborado e apresentados em forma de quadros e tabelas.

**Resultados:**

a análise de 28 estudos selecionados mostrou que a sua maioria era do Brasil, seguidos pela China e Malásia. Quase a metade dos instrumentos validados eram genéricos, e os específicos eram sobre avaliação em diabetes *mellitus* e hanseníase. Os tipos de validação realizados foram de conteúdo e de construto.

**Conclusão:**

há necessidade de construção de instrumentos específicos devido à escassez de estudos sobre o processo de validação de instrumentos para a avaliação da qualidade dos serviços prestados pela atenção primária à saúde em doenças crônicas.

## Destaques

**(1)** Instrumentos validados para a avaliação de serviços em doenças crônicas.

**(2)** Possibilidade de realização de novos estudos de adaptações do PCAT e PACIC.

**(3)** Não foi identificada avaliação para menores de 18 anos com doenças crônicas.

**(4)** Uso de um instrumento genérico para avaliar doenças crônicas específicas.

## Introdução

As doenças crônicas são definidas pela Organização Mundial da Saúde (OMS) como patologias de desenvolvimento lento e que persistem por períodos acima de seis meses, requerendo terapêutica e cuidados mais avançados por um maior período de tempo, estando essas doenças entre as principais causas de mortalidade e morbidade no mundo^1^. 

Os Objetivos de Desenvolvimento Sustentável (ODS) da Organização das Nações Unidas (ONU) reforçam a necessidade de uma maior atenção a agravos como diabetes, tuberculose, hipertensão arterial e hanseníase, necessitando maiores esforços na prevenção e no tratamento dessas doenças, presentes em todas as regiões do mundo^2^.

No cenário brasileiro, o Plano de Ações Estratégicas para o Enfrentamento das Doenças Crônicas e Agravos Não Transmissíveis no Brasil 2021-2030 fortalece as diretrizes para a prevenção dessas doenças e o fortalecimento das ações de promoção da saúde e redução de iniquidades e desigualdades sociais, através da reorganização dos serviços de saúde, estabelecimento de programas e projetos, e investimentos em áreas do conhecimento que favoreçam a pesquisa em gestão, a inovação e a implementação de evidências científicas nos serviços^3^.

A hipertensão arterial sistêmica e o diabetes *mellitus* estão entre as principais doenças crônicas não-transmissíveis. O grande impacto dessas doenças sobre a população brasileira requer a adoção de medidas eficazes e monitoramento apropriado, de modo a oferecer cuidados de saúde capazes de atuar adequadamente nas intervenções e que possam resultar na diminuição da incidência desses agravos, promovendo atividades ligadas à prevenção e ao tratamento farmacológico oportuno^4^.

A hanseníase e a tuberculose são doenças crônicas transmissíveis presentes no território brasileiro. São doenças infectocontagiosas que se configuram como um grande problema de saúde pública^5^. Existe um fator de regionalização relacionado a essas doenças, que apresenta diferentes padrões de distribuição espacial, ligados ao aumento das desigualdades sociais e à existência de bolsões de pobreza. O comportamento dessas doenças pode servir como um indicador de desenvolvimento de uma determinada região, refletindo na necessidade de formulação de políticas públicas, saneamento básico, desenvolvimento econômico e melhor acesso aos serviços de saúde^6^.

Sendo assim, a hanseníase e a tuberculose são doenças prioritárias no cenário nacional, possuindo coordenações específicas que norteiam todo o protocolo de prevenção, promoção à saúde e atendimento nas unidades de Atenção Primária à Saúde, além de serem agravos de interesse das pesquisas pelo Brasil^5^.

A Atenção Primária à Saúde (APS) configura-se como o serviço prioritário e ordenador da rede de atenção para o atendimento das doenças crônicas transmissíveis e não transmissíveis. A APS, no Brasil, está pautada nos princípios norteadores do Sistema Único de Saúde (SUS), e possui a missão de ofertar ações e serviços dentro do conceito de territorialidade^7^. A qualidade em saúde e, principalmente, dos serviços da APS, atualmente se fortalece por representar uma função política e social preponderante, com consequência no processo de transformação e valorização de aspectos e atributos capazes de mensurar a prestação dos serviços ofertados à população^8^.

A APS, nesse cenário, caracteriza-se por um conjunto de ações com o objetivo de desenvolver atenção integral, de qualidade, e que direcione para elevar a autonomia das pessoas e sua situação de saúde de maneira individual e coletiva, por meio da inserção nos serviços de uma atenção em saúde qualificada^9^.

Nesse aspecto, a avaliação de serviços de saúde é essencial como processo de tomada de decisão baseado em evidências científicas capazes de nortear e/ou modificar a prestação de serviços, garantindo uma adequada resposta às demandas de saúde da população, o que permite a reformulação de práticas através da competência gerencial e da incorporação da produção de informação para a definição de novas estratégias de intervenção^10^.

Nessa perspectiva, a construção, adaptação e validação de instrumentos de avaliação de serviços é considerada uma ferramenta gerencial imprescindível na qualidade em saúde^11^. A validação de instrumentos de medidas consiste em criar formulários e questionários que permitam mensurar aquilo a que se propõe, o mais próximo possível da realidade, por meio de propriedades psicométricas e parâmetros que garantam a validade e o uso ampliado destes instrumentos^11^ ^)-(^ ^12^.

Uma busca preliminar no PROSPERO (*International Prospective Register of Systematic Reviews*), MEDLINE (*Medical Literature Analysis and Retrieval System Online)*, CDSR (*Cochrane Database of Systematic Reviews*) e *JBI Evidence Synthesis* (*Joanna Briggs Institute*) foi conduzida para verificação, e nenhuma revisão de escopo ou revisão sistemática atual ou em andamento foi identificada, requerendo a necessidade de realização deste estudo. 

Diante do exposto, este estudo teve como objetivo mapear a literatura cientifica sobre a validação de instrumentos para a avaliação da qualidade dos serviços prestados na atenção primária à saúde em doenças crônicas relacionadas com hipertensão arterial sistêmica, diabetes *mellitus*, hanseníase e tuberculose. 

A presente revisão de escopo tem o intuito de corroborar, a partir de seus achados, os conhecimentos disponíveis sobre a temática em questão, a fim de que possam ser desenvolvidas estratégias mais eficazes no enfrentamento das doenças crônicas, a partir da avaliação dos serviços prestados na atenção primária à saúde, por meio de instrumentos capazes de garantir a confiabilidade e a reprodutibilidade do seu uso. 

## Método

### Tipo de estudo

Trata-se de uma revisão de escopo produzida segundo os critérios e as recomendações estabelecidas pelo JBI^13^ e descrita de acordo com o checklist *Preferred Reporting Items for Systematic Reviews and Meta-Analyses extension for Scoping Reviews* (PRISMA-ScR)^14^. O protocolo deste estudo está publicado na *Open Science Framework*, em: https://osf.io/ynrht/. 

### Cenário de estudo e fontes de informação

As buscas foram realizadas nas seguintes bases de dados: *National Institutes of Health* (PubMed), *Cumulative Index to Nursing and Allied Health Literature* (CINAHL), *Cochrane Central*, EMBASE, Scopus, *Web of Science*, *Psychological Abstracts* (PsyCINFO) e *Leprosy Information Services* (Infolep). A estratégia de busca foi adaptada de acordo com os protocolos adotados por cada base de dados, utilizando uma combinação de vários descritores. A busca por literatura cinza foi realizada no Banco de Teses e Dissertações da Coordenação de Aperfeiçoamento de Pessoal de Nível Superior (CAPES), DART - EBSCO *Open Dissertation*, *Networked Digital Library of Theses and Dissertations* (NDLTD), Biblioteca Digital Brasileira de Teses e Dissertações - (BDTD) - Instituto Brasileiro de Informação em Ciência e Tecnologia (IBICT) e *American Chemical Society* (ACS) *Guide to Scholarly Communication*.

### Período

Este estudo foi realizado de junho a outubro de 2023, seguindo seis passos: critérios de elegibilidade, fontes de informação, busca na literatura, seleção das fontes de evidências, extração de dados e análise e apresentação dos dados.

### População

A revisão foi composta por 4.083 estudos, identificados nas seguintes bases: 2511 na PubMed, 152 na CINAHL, 305 na EMBASE, 324 na Scopus, 108 na *Web of Science*, 21 na PsycINFO, 37 na Infolep, 23 na *Cochrane Library*, 391 no Catálogo de Teses e Dissertações da CAPES, 25 na DART - EBSCO *Open Dissertation*, 21 na *Networked Digital Library of Theses and Dissertations* (NDLTD), 153 na BDTD - Biblioteca Digital Brasileira de Teses e Dissertações - IBICT - Instituto Brasileiro de Informação em Ciência e Tecnologia e 12 na ACS *Guide to Scholarly Communication*. 

### Critérios de seleção

A questão de pesquisa foi elaborada com base no acrônimo PCC (População, Conceito e Contexto)^15^, sendo P: Estudos sobre instrumentos validados para mensurar a qualidade dos serviços de saúde; C: Processo de validação; e C: Atendimento em hipertensão arterial sistêmica, diabetes *mellitus*, hanseníase e tuberculose na atenção primária à saúde. 

Foram incluídos estudos experimentais e quase-experimentais, estudos metodológicos, estudos observacionais analíticos e descritivos, abordagens qualitativas, revisões sistemáticas e metanálises, capítulos de livros, resumos de conferências, teses, dissertações e outras fontes de literatura cinza pertinentes à temática, tais como revistas e sites especializados na área da saúde, sem restrição de idioma e sem recorte temporal. Foram excluídos estudos que avaliaram o autocuidado e estudos que não se encontravam disponíveis na íntegra. Tomando por base o acrônimo PCC, foi formulada a pergunta de pesquisa: Quais as evidências disponíveis na literatura sobre os instrumentos para a avaliação da qualidade dos serviços prestados na atenção primária à saúde em doenças crônicas? 

A pesquisa, neste cenário, em hanseníase, tuberculose, hipertensão arterial e diabetes *mellitus* se justifica por essas doenças serem de interesse das políticas de saúde brasileiras, possuindo ações e diretrizes próprias e específicas que norteiam todos os aspectos de prevenção e promoção à saúde, nas redes de atenção à saúde, do Ministério da Saúde do Brasil.

### Variáveis de estudo

Foi elaborado um instrumento específico para que ocorresse a coleta de dados dos artigos da melhor maneira possível. Este instrumento foi composto pelas seguintes variáveis: tipo de periódico, país de realização do estudo, doença crônica avaliada pelo instrumento, população estudada, tipo de validação adotada, tipo de escala, técnica de validação de conteúdo, técnica de validação de construto, cálculo da consistência interna e cálculo da confiabilidade e identificação dos principais achados no conjunto de evidências.

### Coleta de dados

A estratégia de busca seguiu três etapas^16^. Na primeira, realizou-se uma pesquisa preliminar nas bases PubMed e no *Cumulative Index to Nursing and Allied Health Literature* (CINAHL), com o objetivo de identificar os termos contidos em títulos, resumos e descritores para a montagem da estratégia de busca. Na etapa dois, foi realizada uma segunda pesquisa utilizando os termos encontrados na primeira etapa somados aos descritores identificados. Na terceira etapa, os pesquisadores buscaram nas listas de referências estudos não recuperados nas duas primeiras etapas. 

Foi utilizada uma combinação dos seguintes descritores: Pessoal de Saúde / Health Personnel / Personal de Salud OR Gestor de Saúde / Health Manager / Gestor de Salud OR Pacientes / Patients / Pacientes AND Estudo de Validação / Validation Study / Estudio de Validación AND Qualidade da Assistência à Saúde / Quality of Health Care.

A estratégia de busca completa se encontra descrita na Figura 1, atualizada em 22/08/2023.

**Figura 1:** Estratégia de busca segundo as bases de dados. Cuiabá, MT, Brasil, 2023

Base de dados	Estratégia de busca
PubMed	((“Health Personnel”[Mesh] OR “Health Personnel”) OR (“Health Manager”) OR (“Patients”[Mesh] OR Patients OR Patient)) AND ((“Validation Study” OR “Validation Studies” OR “Validation Tool” OR “Validation Tools” OR “Validation Instruments” OR “Validation Instrument”) AND ((“Health Evaluation”) OR (“Quality of Health Care”[Mesh] OR “Quality of Health Care”))) AND (“Primary Health Care”[Mesh] OR “Primary Health Care” OR “Primary Healthcare” OR “Primary Care”)
CINAHL	(((MH “Health Personnel”) OR “Health Personnel”) OR ((MH “Patients”) OR (Patient OR Patients))) AND ((MH “Validation Studies”) OR (“Validation Study” OR “Validation Studies” OR “Validation Tool” OR “Validation Tools” OR “Validation Instruments” OR “Validation Instrument”)) AND ((“Health Evaluation”) OR ((MH “Quality of Health Care”) OR “Quality of Health Care”)) AND ((MH “Primary Health Care”) OR “Primary Health Care”)
EMBASE	(‘health care personnel’/exp OR ‘health care personnel’ OR ‘patient’/exp OR patient) AND (‘validation study’/exp OR ‘validation study’) AND (‘health care quality’/exp OR ‘health care quality’ OR ‘health evaluation’) AND (‘primary health care’/exp OR ‘primary health care’)
Scopus	TITLE-ABS-KEY ((“Health Personnel” OR “Health Manager” OR Patient) AND (“Validation Study” OR “Validation Studies” OR “Validation Tool” OR “Validation Tools” OR “Validation Instruments” OR “Validation Instrument”) AND (Evaluation OR “Quality of Health”) AND (“Primary Health Care” OR “Primary Healthcare” OR “Primary Care”))
*Web of Science*	(“Health Personnel” OR “Health Manager” OR Patient) AND (“Validation Study” OR “Validation Studies” OR “Validation Tool” OR “Validation Tools” OR “Validation Instruments” OR “Validation Instrument”) AND (Evaluation OR “Quality of Health”) AND (“Primary Health Care” OR “Primary Healthcare” OR “Primary Care”)
PsycINFO	(“Health Personnel” OR “Health Manager” OR Patient) AND (“Validation Study” OR “Validation Studies” OR “Validation Tool” OR “Validation Tools” OR “Validation Instruments” OR “Validation Instrument”) AND (Evaluation OR “Quality of Health”) AND (“Primary Health Care” OR “Primary Healthcare” OR “Primary Care”)
Infolep	(“Validation Study” OR “Validation Studies” OR “Validation Tool” OR “Validation Tools” OR “Validation Instruments” OR “Validation Instrument”)
Cochrane Library	(“Patients” OR “Health Personnel” OR “Health Manager”) AND (“Validation Study” OR “Validation Studies” OR “Validation Tool” OR “Validation Tools” OR “Validation Instruments” OR “Validation Instrument”) AND (“Quality of Health Care” OR Evaluation) AND (“Primary Health Care”) = in Title Abstract Keyword
CAPES Catálogo de Teses e Dissertações	Validação AND Avaliação AND “Atenção Primária à Saúde”
DART - EBSCO *Open Dissertation*	(“Health Personnel” OR “Health Manager” OR Patient) AND (“Validation Study” OR “Validation Studies” OR “Validation Tool” OR “Validation Tools” OR “Validation Instruments” OR “Validation Instrument”) AND (“Primary Health Care” OR “Primary Healthcare” OR “Primary Care”)
*Networked Digital Library of Theses and Dissertations* (NDLTD)	(“Health Personnel” OR “Health Manager” OR Patient) AND (“Validation Study” OR “Validation Studies” OR “Validation Tool” OR “Validation Tools” OR “Validation Instruments” OR “Validation Instrument”) AND (Evaluation OR “Quality of Health”) AND (“Primary Health Care” OR “Primary Healthcare” OR “Primary Care”)
BDTD - Biblioteca Digital Brasileira de Teses e Dissertações - IBICT - Instituto Brasileiro de Informação em Ciência e Tecnologia	Validação AND Avaliação AND “Atenção Primária à Saúde”
*ACS Guide to Scholarly Communication*	(“Health Personnel” OR “Health Manager” OR Patient) AND (“Validation Study” OR “Validation Studies” OR “Validation Tool” OR “Validation Tools” OR “Validation Instruments” OR “Validation Instrument”) AND (“Primary Health Care” OR “Primary Healthcare” OR “Primary Care”)

### Extração de dados

Os estudos foram selecionados por dois revisores independentes, com experiência na temática e na realização de revisão de escopo. Um terceiro revisor foi utilizado para resolver as discordâncias de seleção dos estudos, optando pela inclusão ou exclusão destes.

A primeira seleção ocorreu mediante leitura do título e do resumo, levando-se em consideração a presença de elementos que indicassem ou não que se tratava de um estudo avaliativo, de acordo com as doenças elencadas nos critérios. Em seguida, foram lidos os textos na íntegra e avaliados de acordo com os critérios de inclusão/exclusão. Os textos provenientes de literatura cinza tiveram o mesmo processo de avaliação. 

A extração dos dados ocorreu de acordo com as orientações estabelecidas no manual JBI^13^. A JBI é uma instituição que estabelece normas para a realização das revisões sistemáticas, atualizando dados e fazendo recomendações necessárias para a qualidade desses produtos^12^.

### Tratamento dos dados

Os resultados obtidos foram importados para o programa *EndNote Web*, onde ocorreu a investigação da duplicidade das referências bibliográficas^17^. Para análise, seleção e exclusão dos estudos utilizou-se o *software Rayyan* (*Qatar Computing Research Institute*, Doha, Qatar)^18^, com os mesmos critérios de seleção e exclusão elencados anteriormente, uma vez que a plataforma permitia migrar o arquivo das bases de dados, ficando mais fácil e ágil o tratamento dos dados através dessa ferramenta.

O tratamento dos dados ocorreu de acordo com as orientações estabelecidas no manual JBI^13^. A partir da seleção da amostra final, os resultados foram organizados através de quadros, figuras e tabelas descritivas.

### Aspectos éticos

Por se tratar de um estudo com dados de domínio público e disponíveis na literatura, não houve a necessidade de apreciação por Comitê de Ética em Pesquisa. Contudo, destaca-se que foram respeitados os direitos autorais com as devidas citações de cada autor e periódico. 

## Resultados

Nas 13 bases de dados, identificaram-se 4.083 estudos, sendo 2511 na MEDLINE/PubMed, 152 na CINAHL, 305 na EMBASE, 324 na Scopus, 108 na *Web of Science*, 21 na PsycINFO, 37 na Infolep, 23 na *Cochrane Library*, 391 no Catálogo de Teses e Dissertações da CAPES, 25 na DART - EBSCO *Open Dissertation*, 21 na *Networked Digital Library of Theses and Dissertations* (NDLTD), 153 na BDTD - Biblioteca Digital Brasileira de Teses e Dissertações - IBICT - Instituto Brasileiro de Informação em Ciência e Tecnologia e 12 na ACS *Guide to Scholarly Communication*. Após a seleção por título, resumo e aplicação dos critérios de elegibilidade, 28 estudos^19^ ^-^ ^46^ compuseram a amostra final, conforme fluxograma (Figura 2).

**Figura 2:** Síntese das etapas da revisão sistemática de escopo segundo adaptação do *Preferred Reporting Items for Systematic Reviews and Meta-Analyses*. Cuiabá, MT, Brasil, 2023

Observou-se maior percentual de estudos publicados sem registro de dados quanto ao período de coleta de dados (n=15; 53,6%). Os estudos com registro do período de coleta de dados foram realizados em 2003 e 2016 (n=13; 46,4%). Com relação ao ano de publicação, 6 (21,5%) estudos foram publicados entre 2004 e 2010, 19 entre 2011 e 2020 (67,8%) e 3 (10,7) foram publicados após 2020.

Quanto ao período de coleta de dados, observou-se que uma grande parte dos artigos não continham essa informação, fato este que pode prejudicar a avaliação da qualidade metodológica dos mesmos. A maioria dos artigos foram publicados antes de 2020, o que denota que sua produção retrocede a esta data, devido ao período de avaliação dos mesmos pelo periódico científico. Ambos os fatos merecem destaque, pois podem ser considerados uma limitação quando se avalia a qualidade deste produto e dos manuscritos utilizados.

Quanto ao tipo de periódico, a maioria dos estudos foram publicados em revistas internacionais (n=22; 78,6%). Dentre os países com maior publicação estão o Brasil (n=7; 25,0%), seguido da China (n=3; 10,7%) e da Malásia (n=3; 10,7%). Os estudos apresentaram pelo menos dois ou três tipos diferentes de instrumento de validação, sendo o questionário genérico em sua maioria (n=13, 46,4%). Os questionários eram de avaliações em doenças crônicas em um contexto geral, seguidos de instrumentos para avaliação do diabetes *mellitus* e da hanseníase (n=5; 17,9%). Nessa revisão de escopo, não foram identificados instrumentos para a avaliação da qualidade dos serviços prestados em doenças crônicas para a população de crianças e adolescentes (< de 18 anos). A maioria dos estudos (n=19; 68%) foi realizada em população adulta e idosa (> de 18 anos).

Quanto aos tipos de estudos de validação adotada identificados, constatou-se adaptação transcultural em 50% (n=14) dos estudos; 19 (67,9%) realizaram validação de conteúdo; 18 (64,3%), validação semântica; e 23 (82,1%), validação de construto. Houve cálculo da consistência interna em 92,8 % (n=26) da amostra do estudo, e foi calculada a confiabilidade através do teste/reteste em apenas 13 (46,4%) estudos. Quanto ao tipo de escala adotada, a maior parte dos estudos optou pela escala Likert de 5 pontos (n=16; 57,1%). Os dados acima estão descritos na Tabela 1.

**Tabela 1:** Caracterização da produção científica sobre instrumentos para a avaliação de serviços de saúde em doenças crônicas na Atenção Primária à Saúde (n = 28). Cuiabá, MT, Brasil, 2023

Variáveis	n*	%^†^
**Tipo de revista de publicação**		
Nacional	06	21,4
Internacional	22	78,6
**País de realização do estudo**		
Alemanha	02	07,1
Brasil	07	25,0
China	03	10,7
França	02	07,1
Holanda	02	07,1
Malásia	03	10,7
Outros^‡^	09	32,3
**Doença crônica avaliada pelo instrumento de pesquisa**		
Instrumento genérico^§^	13	46,4
Hipertensão	03	10,7
Diabetes	05	17,9
Hanseníase	05	17,9
Tuberculose	02	07,1
**População estudada**		
< de 18 anos	00	00,0
> de 18 anos	19	68,0
Profissionais de saúde	07	25,0
Gestores	02	07,0
**Tipo de validação adotada** ^||^		
Adaptação transcultural	14	50,0
Validação de conteúdo	19	67,9
Validação de face	06	21,4
Validação semântica	18	64,3
Validação de construto	23	82,1
**Cálculo da consistência interna do instrumento**		
Sim	26	92,9
Não	02	07,1
**Cálculo da confiabilidade do instrumento**		
Sim	13	46,4
Não	15	53,6
**Tipo de escala adotada**		
Dicotômica	01	3,6
Likert de 3 pontos	01	3,6
Likert de 4 pontos	04	14,3
Likert de 5 pontos	16	57,1
Outros^¶^	06	21,4
**Instrumentos identificados**		
Adaptações do PACIC**	08	28,5
Adaptações do PCAT^††^	08	28,5
Outros instrumentos diversos	12	43,0

*N = Número absoluto; ^†^% = Percentual; ^‡^Países com somente um estudo publicado: Canadá, Dinamarca, Espanha, Etiópia, Finlândia, Países Baixos, Turquia, Tailândia e um não descrito; ^§^Utilizado para avaliar doenças crônicas de uma maneira geral, sem especificação; ^||^A maioria dos estudos adota mais de um tipo de validação; ^¶^Não mencionaram o tipo de escala/Likert de 11 pontos; **PACIC = *Patient Assessment of Chronic Illness Care* (PACIC); ^††^PCAT = *Primary Care Assessment Tool*

Com relação à utilização dos instrumentos, observou-se percentuais semelhantes de adaptações tanto para o *Patient Assessment of Chronic Illness Care* (PACIC) (n=8;28,5%) quanto para o *Primary Care Assessment Tool* (PCAT); 43% (n=12) tratavam de outros instrumentos além destes destacados por apresentarem grande percentual.

No que se refere à validação de conteúdo, houve maior percentual de utilização da técnica de grupo focal para análise das questões dos instrumentos (n=11; 57,9%). Nos estudos de validação de construto, 20 deles realizaram análise fatorial (n=23;82,1%), seguidos pela técnica de construto convergente (n=7;30,4%).

No que se refere aos agravos, observou-se maiores percentuais para os instrumentos de diabetes *mellitus* (n=5;17,9%) e hanseníase (n=5;17,9%). Os principais achados para o instrumento sobre diabetes *mellitus* foram: originalidade e adaptação transcultural; continuidade do cuidado, prevenção e promoção da saúde como fenômenos de interesse; população alvo adultos/idosos e profissionais de saúde; técnica de validação de construto divergente e convergente; cálculo do alfa de Cronbach satisfatório como medida de consistência interna e propriedades psicométricas consideradas válidas, estando aptos para serem utilizados em estudos científicos.

Os principais achados para o instrumento sobre hanseníase foram: adaptação do PCAT; população alvo adultos, agentes comunitários de saúde, profissionais de saúde e gestores; validação semântica e validação de construto em espelho a outro instrumento do gênero; cálculo da confiabilidade através do teste/reteste e propriedades psicométricas consideradas válidas, estando aptos para serem utilizados em estudos científicos. Tais achados estão disponibilizados na Figura 3.

**Figura 3:** Síntese das principais evidências encontradas nos estudos de validação de instrumentos para a avaliação de serviços em diabetes e hanseníase (n = 10). Cuiabá, MT, Brasil, 2023

Doença crônica	Principais achados
Diabetes	Instrumentos desenvolvidos originalmente^20^ ^),(^ ^25^ e adaptados transculturalmente^19^ ^),(^ ^30^ ^),(^ ^37^.
	Apresentou a continuidade do cuidado^19^ ^),(^ ^25^, a prevenção e promoção da saúde^20^ ^),(^ ^30^ e o cuidado^37^ como fenômenos de interesse.
	Teve como público-alvo e participantes do estudo a população adulta^25^, adulta e idosa^19^ ^),(^ ^37^ e profissionais de saúde^20^ ^),(^ ^30^.
	Utilizou a técnica de validação de construto convergente/divergente^20^ ^),(^ ^25^ ^),(^ ^37^.
	Apresentou alfa de Cronbach superior a 0,8^19^ ^),(^ ^25^ ^),(^ ^30^ ^),(^ ^37^.
	Os instrumentos apresentaram propriedades psicométricas consideradas válidas, estando aptos para serem utilizados em estudos científicos^19^ ^)-(^ ^20^ ^),(^ ^25^ ^),(^ ^30^ ^),(^ ^37^.
Hanseníase	Os instrumentos foram adaptados do instrumento original denominado *Primary Care Assessment Tool* ^42^ ^)-(^ ^45^.
	Os estudos foram realizados na população adulta^40^ ^),(^ ^44^, com Agentes Comunitários de Saúde^43^, com profissionais de saúde^42^ e com gestores^45^.
	Foram realizadas validação semântica e avaliação das questões quanto à clareza, compreensão e adequação dos itens^40^ ^),(^ ^42^ ^)-(^ ^45^.
	Os instrumentos tiveram o seu construto avaliado através da análise fatorial^43^ ou da técnica de validação em espelho com outro instrumento^42^ ^),(^ ^44^ ^)-(^ ^45^.
	Foi calculada a confiabilidade do instrumento através do teste/reteste^40^ ^),(^ ^42^ ^)-(^ ^45^.
	Os instrumentos apresentaram propriedades psicométricas consideradas válidas, estando aptos para serem utilizados em estudos científicos^40^ ^),(^ ^42^ ^)-(^ ^45^.

## Discussão

O presente estudo identificou escassez de estudos que tratavam sobre o processo de validação de instrumentos para a avaliação da qualidade dos serviços prestados pela APS em doenças crônicas em um contexto geral, o que inclui os instrumentos de avaliação quanto aos agravos para hipertensão arterial, diabetes, hanseníase e tuberculose, o que foi escopo de investigação desta revisão. 

A maioria dos estudos foram publicados em revistas internacionais; produzidos principalmente no Brasil, na China e na Malásia; a maioria dos instrumentos de pesquisa validados era instrumentos genéricos, seguidos de instrumentos específicos para diabetes e hanseníase; os estudos foram realizados em população maior de 18 anos, tendo a validação de conteúdo e a validação de construto como principais processos de validade; houve cálculo de consistência interna na maioria dos estudos e não foi calculada a confiabilidade em nenhum dos estudos; a maioria dos instrumentos possuía escala tipo Likert de 5 pontos, onde a validação de conteúdo ocorreu principalmente através da técnica de grupo focal e a validação de construto através da análise fatorial; os principais instrumentos encontrados foram adaptações do PACIC e PCAT.

Quanto à publicação de estudos em periódicos internacionais (publicações realizadas em revistas de outros países fora do Brasil), existem critérios de classificação para a definição de circulação internacional. Sendo assim, não se pode caracterizar o processo de internacionalização da produção científica tendo por base apenas o país. Essa discussão perpassa também pela indexação do periódico em bases internacionais e pela qualidade da evidência no mundo científico^47^.

A busca incessante pela qualidade dos produtos e pela utilização do método adequado em estudos científicos encontra-se em uma crescente escalada. Assim, conforme a internacionalização se difunde e passa a ser vista como uma meta e um objetivo a ser atingido por autores e instituições, as revistas indexadas em bases de dados internacionais bem conceituadas e com um alto fator de impacto apresentam mais visibilidade e prestígio para atrair e selecionar bons estudos^48^.

Quando se observa que a maioria dos estudos encontrados foram produzidos no Brasil, é preciso refletir sobre o aumento expressivo de vagas e de cursos de mestrado e doutorado no país, gerando pesquisas que necessitam utilizar questionários confiáveis, e que geralmente recorrem aos instrumentos validados ou à realização de estudos de validação^49^. As pesquisas em saúde no Brasil vêm construindo espaços direcionados para a busca de qualidade e aperfeiçoamento de técnicas e melhorias dos estudos científicos, voltando-se principalmente para o uso de tecnologias e avaliações de serviços a partir de diversas perspectivas^50^.

O cuidado com os pacientes com doenças crônicas é um dos principais desafios no mundo atual. É notório que o paciente com uma condição crônica acesse mais os serviços de saúde do que os pacientes que não possuem condições crônicas. Diante desses fatos, ressalta-se a importância da validação de instrumentos genéricos para a avaliação da qualidade da assistência prestada aos pacientes com doenças crônicas na APS.

Vale destacar também o importante papel central da APS no SUS, tratando-se de um modelo de atenção forte e consolidado frente a modelos de outros países. Paralelo a isso, o aumento da cobertura da Atenção Básica e da ESF ao longo dos últimos anos no Brasil também pode ser um fator contribuinte para a crescente demanda por uma saúde baseada em evidências científicas e pela criação de protocolos assistenciais e gerenciais dentro da APS, incentivando a produção de tecnologias e conhecimento nessa área^7^.

A avaliação em hanseníase é de extrema importância para mensurar aspectos que possam impactar na qualidade de vida dos pacientes e na qualidade dos serviços prestados, além de ser uma ferramenta de monitoramento e retorno do funcionamento das ações e percepções dos usuários do serviço^51^. Sendo assim, a hanseníase se mantém como um dos agravos prioritários nas políticas de saúde pública do país.

O diabetes é uma das principais doenças crônicas que acometem a população mundial e está ligado a diversos fatores biológicos e comportamentais. É também uma das principais causas de morte por doenças crônicas^4^. Essas causas fortalecem a necessidade da realização de estudos científicos pautados nesses agravos e, com isso, a utilização de ferramentas de coleta de dados validadas capazes de mensurar realmente aquilo que é proposto.

Com relação ao fato da maioria dos instrumentos de pesquisa nos estudos de validação incluídos terem como público-alvo a população adulta e idosa, sabe-se que a idade é um importante fator de risco para algumas doenças crônicas existentes^52^, o que pode justificar o achado em questão nesta revisão. O Brasil é um país em envelhecimento e com um risco iminente de pessoas com faixa etária mais avançada e que requerem maiores cuidados de saúde e apoio da rede de proteção social^53^, evidenciando a necessidade real de realização de pesquisas que impactem o ciclo das doenças crônicas nessas faixas etárias.

Chama a atenção o fato de não terem sido encontrados instrumentos para a avaliação das doenças crônicas na faixa etária de crianças e adolescentes de uma maneira genérica, e especificamente para as doenças elencadas nesta revisão, a partir dos critérios de elegibilidade adotados. A presença de doenças crônicas nessas faixas etárias pode servir como parâmetro de avaliação para a qualidade e a capacidade de organização das redes de saúde dentro de um sistema integrado^54^.

Nesse ponto, os programas de saúde voltados para crianças e adolescentes na APS vêm apresentando um crescimento e uma busca na aproximação desta clientela dos serviços de saúde, requerendo mecanismos que busquem avaliar o desempenho das ações e serviços de saúde disponibilizados para esse público-alvo. 

O processo de validação de conteúdo de instrumentos é essencial no processo de construção e de seleção dos assuntos abordados, buscando deixar o conteúdo mais confiável e aperfeiçoar o instrumento de medida, de modo que este possa realmente representar a teoria que embasou a etapa de construção e medir o quanto estes itens são capazes de reproduzir o fenômeno proposto^55^. Diante disto, o fato de grande parte dos estudos identificados nesta revisão terem passado pela etapa de validação de conteúdo pode sugerir maior valor ao instrumento produzido. Vale ressaltar também que os estudos que realizaram adaptação transcultural não mencionam a etapa de validação de conteúdo, porém, o instrumento original possivelmente passou por esta etapa.

Observou-se que na maioria dos estudos que descreveram terem utilizado a validação de conteúdo, esta ocorreu através da técnica de grupo focal. Esta técnica se assemelha a uma entrevista em grupo, onde, a partir dos critérios de inclusão e exclusão, são selecionados peritos (especialistas) que se reúnem para avaliar as questões presentes no instrumento de medida e buscam um consenso em grupo^56^.

A maioria dos estudos selecionados nesta revisão passaram por validação de construto através da técnica de análise fatorial. A validação de construto é uma propriedade adotada nos testes psicométricos para avaliar se realmente o instrumento de medida é capaz de medir aquilo a que se propõe, possibilitando, assim, determinar características capazes de explicar as variâncias e o real significado do teste^57^.

Quanto ao construto, faz-se necessário chamar a atenção aos poucos construtos/domínios relativos à saúde mental do público-alvo da avaliação de serviços. Os aspectos inerentes à saúde mental se constituem em uma robusta política de saúde para usuários, profissionais e gestores, com um crescimento que merece destaque através de seus protocolos assistenciais e gerenciais.

O uso da análise fatorial na validação de construto fornece subsídios avaliativos para as possíveis correlações existentes entre as variáveis e suas relações entre si, definidas através de fatores, onde um menor número de traços latentes pode explicar um maior número de variáveis^57^ ^)-(^ ^58^. Assim sendo, os estudos que apresentam o processo de validação de construto, teoricamente, possuem uma maior robustez de propriedades psicométricas válidas, representando melhor o fenômeno estudado, e podendo traduzir a realidade desejada o mais próximo possível.

O construto convergente/divergente diz respeito a uma técnica na qual se utilizam outros instrumentos que medem o mesmo construto teórico e, assim, se avalia se ambos os instrumentos irão apresentar grandezas inversamente ou diretamente proporcionais, permitindo uma validação a partir de conceitos teóricos^59^. A validação em espelho foi utilizada como técnica por meio da qual se adaptou um conjunto de instrumentos a partir do PCAT, e realizou-se a análise fatorial em um deles, e com os demais, onde não foi possível realizar análise fatorial devido ao tamanho da amostra, realizou-se validação em espelho, tendo como referência o instrumento em que ocorreu a análise fatorial^43^ ^),(^ ^45^ ^)-(^ ^46^.

Referente ao cálculo da consistência interna, apenas dois estudos não mencionaram a realização de tal teste. A consistência interna indica se um instrumento é homogêneo ou não. Ela expressa se as subpartes do instrumento são capazes de retratar a medida da mesma característica inerente. Uma consistência interna alta pode indicar que os itens de um instrumento medem o mesmo construto teórico^57^ ^)-(^ ^58^. Assim, o cálculo da consistência interna é essencial no processo de validação de instrumentos de aferição de medidas.

Nesta revisão de escopo foi observado que a maioria dos instrumentos validados não mencionaram o cálculo da confiabilidade através do teste/reteste. Este cálculo mede uma estabilidade temporal do questionário e sua capacidade de reproduzir o mesmo resultado ao longo do tempo e no espaço^57^ ^)-(^ ^58^. Diante disso, se faz necessária e importante a adoção desta medida psicométrica na validação de testes, contribuindo para uma maior qualidade do instrumento de medida, desde que esse instrumento aceite este tipo de medida, uma vez que essa medida psicométrica pode não ser adequada, devido à característica do instrumento, ao tipo de população envolvida ou à ação do tempo.

Nos estudos elencados, o PACIC foi o instrumento mais adaptado. Trata-se de um instrumento que vem sendo testado em diversos países do mundo, pautado no Modelo de Atenção às Condições Crônicas (MACC), e sustentado através de evidências de vários estudos^60^. O instrumento pode ser utilizado de maneira genérica ou para doenças crônicas específicas, evidenciando resultados satisfatórios na avaliação de serviços, sendo considerado de fácil aplicação e de baixo custo^60^.

O PCAT, por sua vez, foi proposto e validado por uma equipe liderada por Barbara Starfield, nos Estados Unidos da América, como uma escala psicométrica para avaliar, através de escores, todos os atributos da APS^9^. A partir de então, diversos autores brasileiros e de outros países começaram a utilizar o PCAT como instrumento para a avaliação da APS, e a realizar processos de adaptação transcultural do instrumento genérico e adaptações específicas para algumas doenças crônicas em particular^61^.

A continuidade do cuidado reflete os conceitos da existência da longitudinalidade, assegurando um cuidado em uma linha temporal, quantas vezes forem necessárias, dimensionando entre si ligações entre as ações de prevenção e de promoção da saúde, refletindo também questões referentes ao acesso aos serviços de saúde^62^. Nas doenças crônicas, a continuidade do cuidado, a prevenção e a promoção da saúde são de extrema importância enquanto ferramentas para o cuidar do usuário e para o direcionamento adequado de ações na APS.

As limitações do estudo são referentes à ausência de clareza na descrição de alguns processos de validação entre os instrumentos dos estudos selecionados, podendo causar possível viés no resultado apresentado. A maioria dos estudos foram publicados antes de 2020, o que denota que sua produção retrocede a esta data, devido ao período de avaliação dos mesmos pelo periódico científico, o que induz, também, a uma limitação quando se avalia a qualidade deste produto e dos manuscritos utilizados.

## Conclusão

Os principais achados se referem à predominância de validação dos instrumentos genéricos e específicos para hanseníase e diabetes, que configuram uma prioridade na atenção primária à saúde em doenças crônicas, e evidenciou-se uma lacuna de conhecimento para instrumentos validados para a atenção em crianças e adolescentes. A maioria dos instrumentos validados eram adaptações do PACIC e do PCAT, demonstrando a importância destes instrumentos na avaliação de doenças crônicas na população adulta e idosa e a partir da percepção de profissionais de saúde e gestores.

As evidências indicam escassez de construção de instrumentos específicos, e ausência de estudos na população infanto-juvenil sobre o processo de validação de instrumentos para a avaliação da qualidade dos serviços prestados pela atenção primária à saúde nas doenças crônicas relacionadas na revisão.

Portanto, recomenda-se a realização de novos estudos, e inclusive a validação de instrumentos específicos para avaliar a qualidade dos serviços perante a cronicidade desses agravos na população de crianças e adolescentes.

# Translation: Instrumentos para evaluar la calidad de los servicios en enfermedades crónicas: revisión de alcance*

**DOI:** 10.1590/1518-8345.7168.4292

**Authors:** Gutembergue Santos de Sousa, Fabiane Verônica da Silva, Fabiana Gulin Longhi, Denise da Costa Boamorte Cortela, Pãmela Rodrigues De Souza Silva, Silvana Margarida Benevides Ferreira

**Affiliations:** 1 Universidade Federal do Mato Grosso, Cuiabá, MT, Brasil.; 2 Universidade de São Paulo, Biblioteca da Faculdade de Enfermagem, São Paulo, SP, Brasil.; 3 Universidade do Estado do Mato Grosso, Faculdade de Medicina, Cáceres, MT, Brasil.

**Keywords:** Estudio de Validación, Evaluación de Programas e Instrumentos de Investigación, Encuestas y Cuestionarios, Enfermedad Crónica, Investigación sobre Servicios de Salud, Métodos

## Abstract

**Objetivo:**

mapear la literatura científica sobre la validación de instrumentos para evaluar la calidad de los servicios prestados en la atención primaria de salud para enfermedades crónicas relacionadas con la hipertensión arterial sistémica, diabetes *mellitus*, lepra y tuberculosis.

**Método:**

revisión de alcance, siguiendo el método del Instituto Joanna Briggs y descrito de acuerdo con el *Preferred Reporting Items for Systematic Reviews and Meta-Analyses Extension for Scoping Reviews*. Se incluyeron 13 bases de datos y literatura gris. La selección de los estudios se realizó previa eliminación de duplicados y evaluación individual y pareada. Los datos se extrajeron basándose en un guión elaborado y se presentaron en cuadros y tablas.

**Resultados:**

el análisis de 28 estudios seleccionados mostró que la mayoría era de Brasil, seguidos de China y Malasia. Casi la mitad de los instrumentos validados eran genéricos, y los específicos cubrían la evaluación de diabetes *mellitus* y lepra. Los tipos de validación realizados fueron de contenido y de constructo.

**Conclusión:**

existe la necesidad de construir instrumentos específicos debido a la escasez de estudios sobre el proceso de validación de instrumentos para evaluar la calidad de los servicios prestados por la atención primaria de salud para enfermedades crónicas.

## Destacados

**(1)** Instrumentos validados para la evaluación de servicios en enfermedades crónicas.

**(2)** Posibilidad de realizar nuevos estudios de adaptaciones del PCAT y PACIC.

**(3)** No se identificó evaluación para niños menores de 18 años con enfermedades crónicas.

**(4)** Uso de un instrumento genérico para evaluar enfermedades crónicas específicas.

## Introducción

Las enfermedades crónicas son definidas por la Organización Mundial de la Salud (OMS) como patologías que se desarrollan lentamente y persisten por períodos superiores a seis meses, requiriendo terapias y cuidados más avanzados durante un período de tiempo más prolongado, estando estas enfermedades entre las principales causas de mortalidad y morbilidad en el mundo^1^. 

Los Objetivos de Desarrollo Sostenible (ODS) de la Organización de las Naciones Unidas (ONU) refuerzan la necesidad de una mayor atención a enfermedades como diabetes, tuberculosis, hipertensión arterial y lepra, requiriendo mayores esfuerzos en la prevención y el tratamiento de estas enfermedades, presentes en todas las regiones del mundo^2^.

En el escenario brasileño, el Plan de Acciones Estratégicas para el Enfrentamiento de las Enfermedades no Transmisibles y Lesiones en Brasil 2021-2030 fortalece los lineamientos para prevenir estas enfermedades y fortalecer las acciones de promoción de la salud y reducción de las inequidades y desigualdades sociales, por medio de la reorganización de los servicios de salud, el establecimiento de programas y proyectos, e inversiones en áreas del conocimiento que favorezcan la investigación en gestión, la innovación y la implementación de la evidencia científica en los servicios^3^.

La hipertensión arterial sistémica y la diabetes *mellitus* se encuentran entre las principales enfermedades crónicas no transmisibles. El gran impacto de estas enfermedades en la población brasileña requiere la adopción de medidas efectivas y un seguimiento apropiado, con el fin de ofrecer cuidados de salud capaces de actuar adecuadamente en las intervenciones y que puedan resultar en una reducción de la incidencia de estas enfermedades, promoviendo actividades vinculadas a prevención y tratamiento farmacológico oportuno^4^.

La lepra y la tuberculosis son enfermedades crónicas transmisibles presentes en el territorio brasileño. Son enfermedades infecciosas que representan un importante problema de salud pública^5^. Existe un factor de regionalización relacionado con estas enfermedades, que presenta diferentes patrones de distribución espacial, vinculados al aumento de las desigualdades sociales y a la existencia de bolsas de pobreza. El comportamiento de estas enfermedades puede servir como indicador del desarrollo de una determinada región, reflejando la necesidad de formular políticas públicas, saneamiento básico, desarrollo económico y mejor acceso a los servicios de salud^6^.

Por lo tanto, la lepra y la tuberculosis son enfermedades prioritarias en el escenario nacional, con coordinación específica que orienta todo el protocolo de prevención, promoción de la salud y atención en las unidades de Atención Primaria de Salud, además de ser enfermedades de interés para la investigación en Brasil^5^.

La Atención Primaria de Salud (APS) es el servicio prioritario y organizador de la red asistencial para la atención de las enfermedades crónicas transmisibles y no transmisibles. La APS, en Brasil, se basa en los principios rectores del *Sistema Único de Saúde* (SUS), y tiene la misión de ofrecer acciones y servicios dentro del concepto de territorialidad^7^. La calidad de la salud y, principalmente, de los servicios de la APS, se fortalece actualmente al representar una función política y social preponderante, con consecuencia en el proceso de transformación y valorización de aspectos y atributos capaces de medir la prestación de los servicios ofrecidos a la población^8^.

La APS, en este escenario, se caracteriza por un conjunto de acciones cuyo objetivo es desarrollar una atención integral y de calidad, y que oriente a incrementar la autonomía de las personas y su situación de salud individual y colectivamente, por medio de la inclusión en servicios de salud calificados^9^.

En este aspecto, la evaluación de los servicios de salud es fundamental como proceso de toma de decisiones basado en evidencia científica capaz de orientar y/o modificar la prestación de servicios, asegurando una respuesta adecuada a las demandas de salud de la población, lo que permite reformular las prácticas a través de la competencia gerencial y la incorporación de la producción de información para definir nuevas estrategias de intervención^10^.

Desde esta perspectiva, la construcción, adaptación y validación de instrumentos de evaluación de servicios se considera una herramienta de gestión esencial en la calidad en salud^11^. La validación de instrumentos de medición consiste en la creación de formularios y cuestionarios que permitan medir lo propuesto, lo más cerca posible de la realidad, por medio de propiedades psicométricas y parámetros que garanticen la validez y uso ampliado de estos instrumentos^11^ ^)-(^ ^12^.

Se realizó una búsqueda preliminar en PROSPERO (*International Prospective Register of Systematic Reviews*), MEDLINE (*Medical Literature Analysis and Retrieval System Online)*, CDSR (*Cochrane Database of Systematic Reviews*) y *JBI Evidence Synthesis* (*Joanna Briggs Institute*) para verificación, y no se identificó una revisión de alcance o revisión sistemática actual o en curso, indicando la necesidad de realizar este estudio. 

Teniendo en cuenta lo anterior, este estudio tuvo como objetivo mapear la literatura científica sobre la validación de instrumentos para evaluar la calidad de los servicios prestados en la atención primaria de salud para enfermedades crónicas relacionadas con la hipertensión arterial sistémica, la diabetes *mellitus*, la lepra y la tuberculosis. 

Esta revisión de alcance tiene como objetivo corroborar, a partir de sus hallazgos, el conocimiento disponible sobre el tema en cuestión, de modo que se puedan desarrollar estrategias más efectivas de enfrentamiento a las enfermedades crónicas, a partir de la evaluación de los servicios prestados en la atención primaria de salud, por medio de instrumentos capaces de garantizar la fiabilidad y reproducibilidad de su uso. 

## Método

### Tipo de estudio

Esta es una revisión de alcance producida de acuerdo con los criterios y recomendaciones establecidos por el manual JBI^13^ y descrita de acuerdo con el *checklist Preferred Reporting Items for Systematic Reviews and Meta-Analyses extension for Scoping Reviews* (PRISMA-ScR)^14^. El protocolo de este estudio está publicado en *Open Science Framework*, en: https://osf.io/ynrht/. 

### Escenario de estudio y fuentes de información

Las búsquedas se realizaron en las siguientes bases de datos: *National Institutes of Health* (PubMed), *Cumulative Index to Nursing and Allied Health Literature* (CINAHL), *Cochrane Central*, EMBASE, Scopus, *Web of Science*, *Psychological Abstracts* (PsyCINFO) y *Leprosy Information Services* (Infolep). La estrategia de búsqueda se adaptó según los protocolos adoptados por cada base de datos, utilizando una combinación de varios descriptores. La búsqueda de literatura gris se realizó en el Banco de Tesis y Disertaciones de la *Coordenação de Aperfeiçoamento de Pessoal de Nível Superior* (CAPES), DART - EBSCO *Open Dissertation*, *Networked Digital Library of Theses and Dissertations* (NDLTD), *Biblioteca Digital Brasileira de Teses e Dissertações* - (BDTD) - *Instituto Brasileiro de Informação em Ciência e Tecnologia* (IBICT) y *American Chemical Society* (ACS) *Guide to Scholarly Communication*.

### Período

Este estudio se llevó a cabo de junio a octubre de 2023, siguiendo seis pasos: criterios de elegibilidad, fuentes de información, búsqueda en la literatura, selección de fuentes de evidencia, extracción de datos y análisis y presentación de los datos.

### Población

La revisión estuvo compuesta por 4.083 estudios, identificados en las siguientes bases de datos: 2511 en PubMed, 152 en CINAHL, 305 en EMBASE, 324 en Scopus, 108 en *Web of Science*, 21 en PsycINFO, 37 en Infolep, 23 en *Cochrane Library*, 391 en *Catálogo de Teses e Dissertações* de CAPES, 25 en DART - EBSCO *Open Dissertation*, 21 en *Networked Digital Library of Theses and Dissertations* (NDLTD), 153 en BDTD - *Biblioteca Digital Brasileira de Teses e Dissertações* **-** IBICT - *Instituto Brasileiro de Informação em Ciência e Tecnologia* y 12 en ACS *Guide to Scholarly Communication*. 

### Criterios de selección

La pregunta de investigación se desarrolló con base en la sigla PCC (Población, Concepto y Contexto)^15^, siendo P: Estudios sobre instrumentos validados para medir la calidad de los servicios de salud; C: Proceso de validación; y C: Atención a hipertensión arterial sistémica, diabetes *mellitus*, lepra y tuberculosis en la atención primaria de salud. 

Se incluyeron estudios experimentales y cuasiexperimentales, estudios metodológicos, estudios observacionales analíticos y descriptivos, enfoques cualitativos, revisiones sistemáticas y metaanálisis, capítulos de libros, resúmenes de congresos, tesis, disertaciones y otras fuentes de literatura gris pertinentes al tema, como revistas y sitios web especializados en el área de la salud, sin restricciones de idioma ni recorte de tiempo. Se excluyeron los estudios que evaluaron el autocuidado y los estudios que no estaban disponibles en su totalidad. A partir de la sigla PCC se formuló la pregunta de investigación: ¿Qué evidencia está disponible en la literatura sobre instrumentos de evaluación de la calidad de los servicios prestados en la atención primaria de salud para enfermedades crónicas? 

La investigación, en este escenario, sobre lepra, tuberculosis, hipertensión arterial y diabetes *mellitus* se justifica porque estas enfermedades son de interés para las políticas de salud brasileñas, teniendo acciones y directrices específicas que orientan todos los aspectos de prevención y promoción de la salud, en las redes de atención de salud del *Ministério de Saúde* de Brasil.

### Variables de estudio

Se desarrolló un instrumento específico para recolectar los datos de los artículos de la mejor manera posible. Este instrumento estuvo compuesto por las siguientes variables: tipo de revista, país en el que se realizó el estudio, enfermedad crónica evaluada por el instrumento, población estudiada, tipo de validación adoptada, tipo de escala, técnica de validación de contenido, técnica de validación de constructo, cálculo de consistencia interna y cálculo de confiabilidad e identificación de los principales hallazgos en el conjunto de evidencias.

### Recolección de datos

La estrategia de búsqueda siguió tres etapas^16^. En la primera, se realizó una búsqueda preliminar en las bases de datos PubMed y en el *Cumulative Index to Nursing and Allied Health Literature* (CINAHL), con el objetivo de identificar los términos contenidos en títulos, resúmenes y descriptores para armar la estrategia de búsqueda. En la etapa dos se realizó una segunda búsqueda utilizando los términos encontrados en la primera etapa agregados a los descriptores identificados. En la tercera etapa, los investigadores buscaron en las listas de referencias estudios no recuperados en las dos primeras etapas. 

Se utilizó una combinación de los siguientes descriptores: Pessoal de Saúde / Health Personnel / Personal de Salud OR Gestor de Saúde / Health Manager / Gestor de Salud OR Pacientes / Patients / Pacientes AND Estudo de Validação / Validation Study / Estudio de Validación AND Qualidade da Assistência à Saúde / Quality of Health Care.

La estrategia de búsqueda completa se describe en la Figura 1, actualizada el 22/08/2023.

**Figura 1:** Estrategia de búsqueda según las bases de datos. Cuiabá, MT, Brasil, 2023

Base de datos	Estrategia de búsqueda
PubMed	((“Health Personnel”[Mesh] OR “Health Personnel”) OR (“Health Manager”) OR (“Patients”[Mesh] OR Patients OR Patient)) AND ((“Validation Study” OR “Validation Studies” OR “Validation Tool” OR “Validation Tools” OR “Validation Instruments” OR “Validation Instrument”) AND ((“Health Evaluation”) OR (“Quality of Health Care”[Mesh] OR “Quality of Health Care”))) AND (“Primary Health Care”[Mesh] OR “Primary Health Care” OR “Primary Healthcare” OR “Primary Care”)
CINAHL	(((MH “Health Personnel”) OR “Health Personnel”) OR ((MH “Patients”) OR (Patient OR Patients))) AND ((MH “Validation Studies”) OR (“Validation Study” OR “Validation Studies” OR “Validation Tool” OR “Validation Tools” OR “Validation Instruments” OR “Validation Instrument”)) AND ((“Health Evaluation”) OR ((MH “Quality of Health Care”) OR “Quality of Health Care”)) AND ((MH “Primary Health Care”) OR “Primary Health Care”)
EMBASE	(‘health care personnel’/exp OR ‘health care personnel’ OR ‘patient’/exp OR patient) AND (‘validation study’/exp OR ‘validation study’) AND (‘health care quality’/exp OR ‘health care quality’ OR ‘health evaluation’) AND (‘primary health care’/exp OR ‘primary health care’)
Scopus	TITLE-ABS-KEY ((“Health Personnel” OR “Health Manager” OR Patient) AND (“Validation Study” OR “Validation Studies” OR “Validation Tool” OR “Validation Tools” OR “Validation Instruments” OR “Validation Instrument”) AND (Evaluation OR “Quality of Health”) AND (“Primary Health Care” OR “Primary Healthcare” OR “Primary Care”))
*Web of Science*	(“Health Personnel” OR “Health Manager” OR Patient) AND (“Validation Study” OR “Validation Studies” OR “Validation Tool” OR “Validation Tools” OR “Validation Instruments” OR “Validation Instrument”) AND (Evaluation OR “Quality of Health”) AND (“Primary Health Care” OR “Primary Healthcare” OR “Primary Care”)
PsycINFO	(“Health Personnel” OR “Health Manager” OR Patient) AND (“Validation Study” OR “Validation Studies” OR “Validation Tool” OR “Validation Tools” OR “Validation Instruments” OR “Validation Instrument”) AND (Evaluation OR “Quality of Health”) AND (“Primary Health Care” OR “Primary Healthcare” OR “Primary Care”)
Infolep	(“Validation Study” OR “Validation Studies” OR “Validation Tool” OR “Validation Tools” OR “Validation Instruments” OR “Validation Instrument”)
*Cochrane Library*	(“Patients” OR “Health Personnel” OR “Health Manager”) AND (“Validation Study” OR “Validation Studies” OR “Validation Tool” OR “Validation Tools” OR “Validation Instruments” OR “Validation Instrument”) AND (“Quality of Health Care” OR Evaluation) AND (“Primary Health Care”) = in Title Abstract Keyword
*CAPES Catálogo de Teses e Dissertações*	Validação AND Avaliação AND “Atenção Primária à Saúde”
DART - EBSCO *Open Dissertation*	(“Health Personnel” OR “Health Manager” OR Patient) AND (“Validation Study” OR “Validation Studies” OR “Validation Tool” OR “Validation Tools” OR “Validation Instruments” OR “Validation Instrument”) AND (“Primary Health Care” OR “Primary Healthcare” OR “Primary Care”)
*Networked Digital Library of Theses and Dissertations* (NDLTD)	(“Health Personnel” OR “Health Manager” OR Patient) AND (“Validation Study” OR “Validation Studies” OR “Validation Tool” OR “Validation Tools” OR “Validation Instruments” OR “Validation Instrument”) AND (Evaluation OR “Quality of Health”) AND (“Primary Health Care” OR “Primary Healthcare” OR “Primary Care”)
BDTD - *Biblioteca Digital Brasileira de Teses e Dissertações* - IBICT - *Instituto Brasileiro de Informação em Ciência e Tecnologia*	Validação AND Avaliação AND “Atenção Primária à Saúde”
*ACS Guide to Scholarly Communication*	(“Health Personnel” OR “Health Manager” OR Patient) AND (“Validation Study” OR “Validation Studies” OR “Validation Tool” OR “Validation Tools” OR “Validation Instruments” OR “Validation Instrument”) AND (“Primary Health Care” OR “Primary Healthcare” OR “Primary Care”)

### Extracción de datos

Los estudios fueron seleccionados por dos revisores independientes, con experiencia en el tema y en la realización de revisión de alcance. Se utilizó un tercer revisor para resolver los desacuerdos respecto a la selección de los estudios, optando por su inclusión o exclusión.

La primera selección ocurrió mediante la lectura del título y del resumen, teniendo en cuenta la presencia de elementos que indicaban o no que se trataba de un estudio evaluativo, según las enfermedades enumeradas en los criterios. Luego, los textos fueron leídos íntegramente y evaluados según los criterios de inclusión/exclusión. Los textos de la literatura gris tuvieron el mismo proceso de evaluación. 

La extracción de datos se produjo de acuerdo con las pautas establecidas en el manual JBI^13^. JBI es una institución que establece estándares para realizar revisiones sistemáticas, actualizar datos y hacer recomendaciones necesarias para la calidad de estos productos^12^.

### Procesamiento de los datos

Los resultados obtenidos fueron importados al programa *EndNote Web*, donde se realizó la investigación de la duplicidad de las referencias bibliográficas^17^. Para el análisis, selección y exclusión de estudios se utilizó el *software Rayyan* (*Qatar Computing Research Institute*, Doha, Qatar)^18^, con los mismos criterios de selección y exclusión enumerados anteriormente, ya que la plataforma permitía migrar el archivo de las bases de datos, haciendo que el procesamiento de los datos fuera más fácil y ágil por medio de esta herramienta.

El procesamiento de datos se realizó de acuerdo con los lineamientos establecidos en el manual del JBI^13^. Luego de seleccionar la muestra final, los resultados se organizaron a través de cuadros, figuras y tablas descriptivas.

### Aspectos éticos

Como se trata de un estudio con datos de dominio público y disponibles en la literatura, no fue necesaria la consideración por parte de un Comité de Ética en Investigación. Sin embargo, cabe señalar que se respetaron los derechos de autor con las debidas citaciones a cada autor y periódico. 

## Resultados

En las 13 bases de datos se identificaron 4.083 estudios, de los cuales 2511 estaban en MEDLINE/PubMed, 152 en CINAHL, 305 en EMBASE, 324 en Scopus, 108 en *Web of Science*, 21 en PsycINFO, 37 en Infolep, 23 en *Cochrane Library*, 391 en *Catálogo de Teses e Dissertações* de CAPES, 25 en DART - EBSCO *Open Dissertation*, 21 na *Networked Digital Library of Theses and Dissertations* (NDLTD), 153 en BDTD - *Biblioteca Digital Brasileira de Teses e Dissertações* - IBICT - *Instituto Brasileiro de Informação em Ciência e Tecnologia* y 12 en ACS *Guide to Scholarly Communication*. Después de la selección por título, resumen y aplicación de criterios de elegibilidad, 28 estudios^19^ ^)-(^ ^46^ compusieron la muestra final, según el flujograma (Figura 2).

**Figura 2:** Resumen de las etapas de la revisión sistemática de alcance según adaptación del *Preferred Reporting Items for Systematic Reviews and Meta-Analyses*. Cuiabá, MT, Brasil, 2023

Se observó un mayor porcentaje de estudios publicados sin registro de datos respecto al período de recolección de datos (n=15; 53,6%). Los estudios con registro del periodo de recolección de datos se realizaron en 2003 y 2016 (n=13; 46,4%). En cuanto al año de publicación, 6 (21,5%) estudios fueron publicados entre 2004 y 2010, 19 entre 2011 y 2020 (67,8%) y 3 (10,7) fueron publicados después de 2020.

En cuanto al período de recolección de datos, se observó que gran parte de los artículos no contenían esta información, hecho que podría perjudicar la evaluación de su calidad metodológica. La mayoría de los artículos fueron publicados antes del año 2020, lo que indica que su producción se remonta a esta fecha, debido al período de evaluación de los artículos por parte de la revista científica. Ambos hechos merecen ser resaltados, ya que pueden considerarse una limitación a la hora de evaluar la calidad de este producto y de los manuscritos utilizados.

En cuanto al tipo de revista, la mayoría de los estudios fueron publicados en revistas internacionales (n=22; 78,6%). Entre los países con mayores publicaciones se encuentran Brasil (n=7; 25,0%), seguido de China (n=3; 10,7%) y Malasia (n=3; 10,7%). Los estudios presentaron al menos dos o tres tipos diferentes de instrumentos de validación, la mayoría de los cuales fueron cuestionarios genéricos (n=13, 46,4%). Los cuestionarios eran para evaluaciones de enfermedades crónicas en un contexto general, seguidos de instrumentos para evaluar diabetes *mellitus* y lepra (n=5; 17,9%). En esta revisión de alcance, no se identificaron instrumentos para evaluar la calidad de los servicios prestados en enfermedades crónicas para la población de niños y adolescentes (< 18 años). La mayoría de los estudios (n=19; 68%) se realizaron en población adulta y de adultos mayores (> 18 años).

Respecto a los tipos de estudios de validación identificados, la adaptación transcultural se encontró en el 50% (n=14) de los estudios; 19 (67,9%) realizaron validación de contenido; 18 (64,3%), validación semántica; y 23 (82,1%), validación de constructo. La consistencia interna se calculó en el 92,8% (n=26) de la muestra del estudio, y la confiabilidad se calculó mediante test/retest en solamente 13 (46,4%) estudios. En cuanto al tipo de escala adoptada, la mayoría de los estudios optaron por la escala Likert de 5 puntos (n=16; 57,1%). Los datos anteriores se describen en la Tabla 1.

**Tabla 1:** Caracterización de la producción científica sobre instrumentos para la evaluación de servicios de salud en enfermedades crónicas en la Atención Primaria de Salud (n = 28). Cuiabá, MT, Brasil, 2023

Variables	n*	%^†^
**Tipo de revista de publicación**		
Nacional	06	21,4
Internacional	22	78,6
**País de realización del estudio**		
Alemania	02	07,1
Brasil	07	25,0
China	03	10,7
Francia	02	07,1
Países Bajos	02	07,1
Malasia	03	10,7
Otros^‡^	09	32,3
**Enfermedad crónica evaluada por el instrumento de investigación**		
Instrumento genérico^§^	13	46,4
Hipertensión	03	10,7
Diabetes	05	17,9
Lepra	05	17,9
Tuberculosis	02	07,1
**Población estudiada**		
< de 18 años	00	00,0
> de 18 años	19	68,0
Profesionales de la salud	07	25,0
Gestores	02	07,0
**Tipo de validación adoptada** ^||^		
Adaptación transcultural	14	50,0
Validación de contenido	19	67,9
Validación aparente	06	21,4
Validación semántica	18	64,3
Validación de constructo	23	82,1
**Cálculo de la consistencia interna del instrumento**		
Sí	26	92,9
No	02	07,1
**Cálculo de la confiabilidad del instrumento**		
Sí	13	46,4
No	15	53,6
**Tipo de escala adoptada**		
Dicotómica	01	3,6
Likert de 3 puntos	01	3,6
Likert de 4 puntos	04	14,3
Likert de 5 puntos	16	57,1
Otros^¶^	06	21,4
**Instrumentos identificados**		
Adaptaciones del PACIC**	08	28,5
Adaptaciones del PCAT^††^	08	28,5
Otros instrumentos diversos	12	43,0

*N = Número absoluto; ^†^% = Porcentaje; ^‡^Países con un solo estudio publicado: Canadá, Dinamarca, España, Etiopía, Finlandia, Países Bajos, Turquía, Tailandia y uno no descrito; ^§^Utilizado para evaluar enfermedades crónicas en general, sin especificación; ^||^La mayoría de los estudios adoptan más de un tipo de validación; ^¶^No mencionaron el tipo de escala/Likert de 11 puntos; **PACIC = *Patient Assessment of Chronic Illness Care* (PACIC); ^††^PCAT = *Primary Care Assessment Tool*

En cuanto al uso de los instrumentos, se observaron porcentajes similares de adaptaciones tanto para el *Patient Assessment of Chronic Illness Care* (PACIC) (n=8;28,5%) como para el *Primary Care Assessment Tool* (PCAT); 43% (n=12) se ocupó de instrumentos distintos a los destacados como de gran porcentaje.

Con respecto a la validación de contenido, hubo mayor porcentaje de uso de la técnica de grupo focal para analizar las preguntas de los instrumentos (n=11; 57,9%). En los estudios de validación de constructo, 20 de ellos realizaron análisis factorial (n=23;82,1%), seguidos de la técnica de constructo convergente (n=7;30,4%).

En cuanto a las enfermedades, se observaron mayores porcentajes para los instrumentos de diabetes *mellitus* (n=5;17,9%) y lepra (n=5;17,9%). Los principales hallazgos del instrumento sobre diabetes *mellitus* fueron: originalidad y adaptación transcultural; continuidad del cuidado, prevención y promoción de la salud como fenómenos de interés; población objetivo adultos/adultos mayores y profesionales de la salud; técnica de validación de constructo divergente y convergente; cálculo del alfa de Cronbach satisfactorio como medida de consistencia interna y propiedades psicométricas consideradas válidas, pudiendo ser utilizadas en estudios científicos.

Los principales hallazgos del instrumento sobre lepra fueron: adaptación del PCAT; población objetivo adultos, agentes comunitarios de salud, profesionales de la salud y gestores; validación semántica y validación de constructo reflejando otro instrumento de su tipo; cálculo de confiabilidad mediante test/retest y propiedades psicométricas consideradas válidas, siendo adecuados para su uso en estudios científicos. Estos hallazgos están disponibles en la Figura 3.

**Figura 3:** Síntesis de las principales evidencias encontradas en estudios de validación de instrumentos para la evaluación de servicios en diabetes y lepra (n = 10). Cuiabá, MT, Brasil, 2023

Enfermedad crónica	Principales hallazgos
Diabetes	Instrumentos desarrollados originalmente^20^ ^),(^ ^25^ y adaptados transculturalmente^19^ ^),(^ ^30^ ^),(^ ^37^.
	Presentó la continuidad del cuidado^19^ ^),(^ ^25^, la prevención y promoción de la salud^20^ ^),(^ ^30^ y el cuidado^37^ como fenómenos de interés.
	El público objetivo y los participantes del estudio fueron la población adulta^25^, adulta mayor^19^ ^),(^ ^37^ y profesionales de la salud^20^ ^),(^ ^30^.
	Se utilizó la técnica de validación de constructo convergente/divergente^20^ ^),(^ ^25^ ^),(^ ^37^.
	Presentó alfa de Cronbach mayor a 0,8^19^ ^),(^ ^25^ ^),(^ ^30^ ^),(^ ^37^.
	Los instrumentos presentaron propiedades psicométricas consideradas válidas, siendo adecuados para su uso en estudios científicos^19^ ^)-(^ ^20^ ^),(^ ^25^ ^),(^ ^30^ ^),(^ ^37^.
Lepra	Los instrumentos fueron adaptados del instrumento original denominado *Primary Care Assessment Tool* ^42^ ^)-(^ ^45^.
	Los estudios fueron realizados en población adulta^40^ ^),(^ ^44^, con los Agentes Comunitarios de Salud^43^, con profesionales de la salud^42^ y con gestores^45^.
	Se realizó validación semántica y evaluación de preguntas en cuanto a claridad, comprensión y adecuación de los ítems^40^ ^),(^ ^42^ ^)-(^ ^45^.
	Los instrumentos tuvieron su constructo evaluado mediante análisis factorial^43^ o la técnica de validación espejo con otro instrumento^42^ ^),(^ ^44^ ^)-(^ ^45^.
	La confiabilidad del instrumento se calculó mediante test/retest^40^ ^),(^ ^42^ ^)-(^ ^45^.
	Los instrumentos presentaron propiedades psicométricas consideradas válidas, siendo adecuados para su uso en estudios científicos^40^ ^),(^ ^42^ ^)-(^ ^45^.

## Discusión

El presente estudio identificó una falta de estudios que abordaran el proceso de validación de instrumentos para evaluar la calidad de los servicios prestados por la APS en enfermedades crónicas en un contexto general, lo que incluye instrumentos de evaluación sobre problemas como hipertensión arterial, diabetes, lepra y tuberculosis, que fue el alcance de la investigación de esta revisión. 

La mayoría de los estudios se publicaron en revistas internacionales; producidos principalmente en Brasil, China y Malasia; la mayoría de los instrumentos de investigación validados fueron genéricos, seguidos de instrumentos específicos para diabetes y lepra; los estudios se realizaron en población mayor de 18 años, teniendo como principales procesos de validez la validación de contenido y la validación de constructo; hubo cálculo de consistencia interna en la mayoría de los estudios y no se calculó la confiabilidad en ninguno de los estudios; la mayoría de los instrumentos contó con una escala Likert de 5 puntos, donde la validación de contenido ocurrió principalmente mediante la técnica de grupos focales y la validación de constructo mediante análisis factorial; los principales instrumentos encontrados fueron adaptaciones del PACIC y PCAT.

En cuanto a la publicación de estudios en revistas internacionales (publicaciones realizadas en revistas de otros países fuera de Brasil), existen criterios de clasificación para definir circulación internacional. Por tanto, el proceso de internacionalización de la producción científica no puede caracterizarse únicamente en función del país. Esta discusión abarca también la indexación de la revista en bases de datos internacionales y la calidad de la evidencia en el mundo científico^47^.

La búsqueda incesante de la calidad de los productos y del uso del método adecuado en los estudios científicos es cada vez mayor. Así, a medida que la internacionalización se extiende y comienza a ser vista como una meta y un objetivo a alcanzar por autores e instituciones, las revistas indexadas en bases de datos internacionales reconocidas y con un alto factor de impacto tienen más visibilidad y prestigio para atraer y seleccionar buenos estudios^48^.

Cuando se observa que la mayoría de los estudios encontrados fueron producidos en Brasil, es necesario reflexionar sobre el aumento significativo de vacantes y cursos de maestría y doctorado en el país, generando investigaciones que necesitan el uso de cuestionarios confiables, y que generalmente recurren a instrumentos validados o la realización de estudios de validación^49^. La investigación en salud en Brasil viene construyendo espacios orientados a la búsqueda de la calidad y perfeccionamiento de las técnicas y mejoras en los estudios científicos, centrándose principalmente en el uso de tecnologías y evaluaciones de servicios desde diferentes perspectivas^50^.

El cuidado con pacientes con enfermedades crónicas es uno de los principales desafíos del mundo actual. Es notable que el paciente con una condición crónica acceda más a los servicios de salud que los pacientes que no tienen condiciones crónicas. Ante estos hechos, se destaca la importancia de validar instrumentos genéricos para evaluar la calidad de la atención brindada a pacientes con enfermedades crónicas en la APS.

También vale la pena resaltar el importante papel central de la APS en el SUS, por ser un modelo de atención fuerte y consolidado frente a modelos de otros países. Paralelamente, el aumento de la cobertura de la Atención Primaria y del ESF en los últimos años en Brasil también puede ser un factor que contribuya a la creciente demanda de atención de salud basada en evidencia científica y a la creación de protocolos de atención y gestión en la APS, incentivando la producción de tecnologías y conocimiento en esta área^7^.

La evaluación en lepra es de suma importancia para medir aspectos que pueden impactar la calidad de vida de los pacientes y la calidad de los servicios brindados, además de ser una herramienta de seguimiento y retroalimentación sobre el funcionamiento de las acciones y percepciones de los usuarios del servicio^51^. Por tanto, la lepra sigue siendo una de las enfermedades prioritarias en las políticas de salud pública del país.

La diabetes es una de las principales enfermedades crónicas que afectan a la población mundial y está asociada a varios factores biológicos y comportamentales. También es una de las principales causas de muerte por enfermedades crónicas^4^. Estas causas fortalecen la necesidad de realizar estudios científicos basados en estos problemas y, con ello, el uso de herramientas de recolección de datos validadas y capaces de medir realmente lo propuesto.

Respecto al hecho de que la mayoría de los instrumentos de investigación en los estudios de validación incluidos se dirigen a la población adulta y adulta mayor, se sabe que la edad es un factor de riesgo importante para algunas enfermedades crónicas existentes^52^, lo que puede justificar el hallazgo en cuestión en esta revisión. Brasil es un país en proceso de envejecimiento y con riesgo inminente de que las personas mayores requieran más cuidados de salud y apoyo de la red de protección social^53^, destacando la necesidad real de realizar investigaciones que impacten el ciclo de las enfermedades crónicas en esos grupos de edad.

Es de destacar que no se encontraron instrumentos para evaluar las enfermedades crónicas en el grupo etario de niños y adolescentes de forma genérica, y específicamente para las enfermedades enumeradas en esta revisión, con base en los criterios de elegibilidad adoptados. La presencia de enfermedades crónicas en estos grupos de edad puede servir como parámetro de evaluación de la calidad y capacidad de organización de las redes de salud dentro de un sistema integrado^54^.

 En este punto, los programas de salud dirigidos a niños y adolescentes en la APS vienen mostrando un crecimiento y una búsqueda por acercar esta clientela a los servicios de salud, requiriendo mecanismos que busquen evaluar el desempeño de las acciones y servicios de salud puestos a disposición de este público objetivo. 

El proceso de validación del contenido del instrumento es fundamental en el proceso de construcción y selección de los temas tratados, buscando hacer más confiable el contenido y perfeccionar el instrumento de medición, para que pueda representar verdaderamente la teoría que sustentó la construcción y calcular en qué medida estos ítems son capaces de reproducir el fenómeno propuesto^55^. Ante esto, el hecho de que la mayoría de los estudios identificados en esta revisión hayan pasado la etapa de validación de contenido puede sugerir un mayor valor para el instrumento elaborado. También cabe resaltar que los estudios que realizaron adaptación transcultural no mencionan la etapa de validación de contenido, sin embargo, el instrumento original posiblemente pasó por esta etapa.

Se observó que en la mayoría de los estudios que describieron haber utilizado la validación de contenido, esto ocurrió a través de la técnica del grupo focal. Esta técnica es similar a una entrevista grupal, donde, a partir de los criterios de inclusión y exclusión, se seleccionan expertos (especialistas) que se reúnen para evaluar las cuestiones presentes en el instrumento de medición y buscar un consenso grupal^56^.

La mayoría de los estudios seleccionados en esta revisión se sometieron a validación de constructo mediante la técnica de análisis factorial. La validación de constructo es una propiedad adoptada en las pruebas psicométricas para evaluar si el instrumento de medición es realmente capaz de medir lo que se pretende medir, permitiendo determinar características capaces de explicar las varianzas y el significado real de la prueba^57^.

En cuanto al constructo, es necesario llamar la atención sobre los pocos constructos/dominios relacionados con la salud mental del público objetivo de la evaluación de los servicios. Los aspectos inherentes a la salud mental constituyen una política de salud robusta para usuarios, profesionales y gestores, con un crecimiento que merece ser destacado a través de sus protocolos de atención y gestión.

El uso del análisis factorial en la validación de constructo brinda apoyo evaluativo a posibles correlaciones entre las variables y sus relaciones entre sí, definidas a través de factores, donde un menor número de rasgos latentes pueden explicar un mayor número de variables^57^ ^)-(^ ^58^. Por tanto, los estudios que presentan el proceso de validación de constructo, teóricamente, tienen mayor robustez de propiedades psicométricas válidas, representando mejor el fenómeno estudiado, y traduciendo la realidad deseada lo más fielmente posible.

El constructo convergente/divergente se refiere a una técnica en la que se utilizan otros instrumentos que miden el mismo constructo teórico y, así, se evalúa si ambos instrumentos presentarán cantidades inversa o directamente proporcionales, permitiendo la validación con base en conceptos teóricos^59^. Se utilizó la validación espejo como técnica mediante la cual se adaptó un conjunto de instrumentos del PCAT, y se realizó análisis factorial con uno de ellos, y con los demás, donde no fue posible realizar análisis factorial debido al tamaño de la muestra, se realizó validación espejo, tomando como referencia el instrumento en el que ocurrió el análisis factorial^43^ ^),(^ ^45^ ^)-(^ ^46^.

En cuanto al cálculo de la consistencia interna, solamente dos estudios no mencionaron la realización de dicha prueba. La consistencia interna indica si un instrumento es homogéneo o no. Expresa si las subpartes del instrumento son capaces de reflejar la medida de una misma característica inherente. Una alta consistencia interna puede indicar que los ítems de un instrumento miden el mismo constructo teórico^57^ ^)-(^ ^58^. Por tanto, el cálculo de la consistencia interna es fundamental en el proceso de validación de instrumentos de medida.

En esta revisión de alcance, se observó que la mayoría de los instrumentos validados no mencionaron el cálculo de la confiabilidad mediante test/retest. Este cálculo mide la estabilidad temporal del cuestionario y su capacidad para reproducir el mismo resultado en el tiempo y el espacio^57^ ^)-(^ ^58^. Ante esto, es necesario e importante adoptar esta medida psicométrica en la validación de pruebas, contribuyendo a una mayor calidad del instrumento de medición, siempre y cuando este instrumento acepte este tipo de medida, ya que esta medida psicométrica puede no ser la adecuada, debido a las características del instrumento, al tipo de población involucrada o a los efectos del tiempo.

De los estudios listados, el PACIC fue el instrumento más adaptado. Se trata de un instrumento que ha sido probado en varios países del mundo, basado en el Modelo de Atención de las Condiciones Crónicas (MACC), y respaldado por evidencia de varios estudios^60^. El instrumento puede ser utilizado de forma genérica o para enfermedades crónicas específicas, mostrando resultados satisfactorios en la evaluación de servicios, considerándose de fácil aplicación y bajo costo^60^.

El PCAT, por su parte, fue propuesto y validado por un equipo liderado por Barbara Starfield, en Estados Unidos de América, como una escala psicométrica para evaluar, a través de puntuaciones, todos los atributos de la APS^9^. A partir de entonces, varios autores brasileños y de otros países comenzaron a utilizar el PCAT como instrumento de evaluación de la APS, y a realizar procesos de adaptación transcultural del instrumento genérico y adaptaciones específicas para algunas enfermedades crónicas en particular^61^.

La continuidad del cuidado refleja los conceptos de existencia de la longitudinalidad, asegurando el cuidado en una línea de tiempo, tantas veces como sea necesario, dimensionando los vínculos entre las acciones de prevención y de promoción de la salud, reflejando también cuestiones relativas al acceso a los servicios de salud^62^. En las enfermedades crónicas, la continuidad del cuidado, la prevención y la promoción de la salud son de suma importancia como herramientas para cuidar al usuario y orientar adecuadamente las acciones en la APS.

Las limitaciones del estudio se refieren a la falta de claridad en la descripción de algunos procesos de validación entre los instrumentos de los estudios seleccionados, lo que puede provocar posibles sesgos en los resultados presentados. La mayoría de los estudios fueron publicados antes de 2020, lo que indica que su producción se remonta a esta fecha, debido al período de evaluación de los mismos por parte de la revista científica, lo que también conlleva una limitación a la hora de evaluar la calidad de este producto y de los manuscritos utilizados.

## Conclusión

Los principales hallazgos se refieren al predominio de la validación de instrumentos genéricos y específicos para lepra y diabetes, que constituyen una prioridad en la atención primaria de salud en enfermedades crónicas, y se destacó un vacío de conocimiento sobre instrumentos validados para la atención a niños y adolescentes. La mayoría de los instrumentos validados fueron adaptaciones del PACIC y PCAT, demostrando la importancia de estos instrumentos en la evaluación de enfermedades crónicas en la población adulta y adulta mayor y basados en la percepción de profesionales y gestores de salud.

Las evidencias indican falta de construcción de instrumentos específicos, y ausencia de estudios en población infantil y adolescente sobre el proceso de validación de instrumentos para evaluar la calidad de los servicios prestados por la atención primaria de salud en las enfermedades crónicas enumeradas en la revisión.

Por tanto, se recomienda la realización de nuevos estudios, e incluso la validación de instrumentos específicos para evaluar la calidad de los servicios dada la cronicidad de estas enfermedades en la población de niños y adolescentes.
